# Harnessing Plant’s Arsenal: Essential Oils as Promising Tools for Sustainable Management of Potato Late Blight Disease Caused by *Phytophthora infestans*—A Comprehensive Review

**DOI:** 10.3390/molecules28217302

**Published:** 2023-10-27

**Authors:** Florian Martini, M. Haïssam Jijakli, Eric Gontier, Jérôme Muchembled, Marie-Laure Fauconnier

**Affiliations:** 1 Joint and Research Unit, 1158 BioEcoAgro Junia, 59000 Lille, France; jerome.muchembled@junia.com; 2 Laboratory of Chemistry of Natural Molecules, Gembloux Agro-Bio Tech, Liege University, Passage des Déportés 2, 5030 Gembloux, Belgium; marie-laure.fauconnier@uliege.be; 3 Laboratory of Plant Biology and Innovation, BIOPI-UPJV, UMRT BioEcoAgro INRAE1158, UFR Sciences of University of Picardie Jules Verne, 33 rue Saint Leu, 80000 Amiens, France; eric.gontier@u-picardie.fr; 4 Integrated and Urban Plant Pathology Laboratory, Gembloux Agro-Bio Tech, Liege University, Passage des Déportés 2, 5030 Gembloux, Belgium; mh.jijakli@uliege.be

**Keywords:** *Phytophthora infestans*, potato late blight disease, oomycete, essential oil, terpenoids, phenylpropanoids, mechanisms of action, cell membrane

## Abstract

Potato late blight disease is caused by the oomycete *Phytophthora infestans* and is listed as one of the most severe phytopathologies on Earth. The current environmental issues require new methods of pest management. For that reason, plant secondary metabolites and, in particular, essential oils (EOs) have demonstrated promising potential as pesticide alternatives. This review presents the up-to-date work accomplished using EOs against *P. infestans* at various experimental scales, from in vitro to in vivo. Additionally, some cellular mechanisms of action on *Phytophthora* spp., especially towards cell membranes, are also presented for a better understanding of anti-oomycete activities. Finally, some challenges and constraints encountered for the development of EOs-based biopesticides are highlighted.

## 1. Introduction

Potato (*Solanum tuberosum* L., 1753) is recognized as the third most significant crop for global human consumption [1]. With an annual production exceeding 350 million tons harvested over an estimated area of 19 million hectares [1,2], it holds the top position among non-cereal crops in terms of yield [3]. The versatility of potato in human diets, coupled with its high edible biomass reaching up to 80% [2], makes it a vital contributor to food security across the globe [1]. Indeed, *S. tuberosum* gained success in the food habits of numerous populations thanks to both the facilities of its cultivation [4,5] and significant source of energy and essential metabolites (macro and micronutrients) it provides [6,7]. In a world where the population is projected to exceed 9 billion people by 2050 [8], meeting the increased demand for high-quality food will be crucial, and potato will definitely play a major role. Given these reasons, efforts focusing on the management of its pests, including late blight disease, have become strongly promoted topics.

*Phytophthora infestans* (Mont.) de Bary, 1876, is generally recognized as the worst pathogen of potato [9]. The first strains originally came from Central America, more specifically, from the Toluca Valley, Mexico [10,11]. After spreading across the United States, they migrated to Europe and eventually expanded worldwide. In fact, potato late blight caused the devastating Irish famine in the 1850s, resulting in the deaths of over 1 million people and forcing many others to migrate from Ireland [12]. This event spurred scientists to start studying plant diseases, leading to the birth of phytopathology as a scientific field on its own [13]. From now on, in order to effectively combat a plant pathogen, it is crucial to accurately describe it. This requires a thorough understanding of both its taxonomy and biology.

The genus *Phytophthora* encompasses over one hundred species [14]. The majority have been identified as plant pathogens [15] causing various diseases around the world. They belong to the clade of oomycetes; these are eukaryotic microorganisms, part of the kingdom of the Chromista [16,17]. They are usually referred to as “pseudo-fungi” because of some shared similarities they exhibit with fungi, such as the mode of nutrition and comparable morphology [18]. Nonetheless, oomycetes phylogenetically diverged from Eumycetes and differ notably by the content of their cell wall (cellulose instead of chitin) [19,20].

Among those, *Phytophthora infestans* was probably the first species to be observed and classified. It is commonly known to cause both potato and tomato late blight disease [21,22]. Potato late blight is widely recognized as the most severe and problematic disease affecting potatoes. It does not only affect the foliage of potato plants but also the tubers, both before and after harvest [23]. When the environmental conditions are suitable for its optimal development (i.e., relative humidity superior to 90% and temperature between 15 et 25 °C) [24], late blight can devastate a whole field of potato within a matter of days [25,26]. As a consequence, the annual costs associated with both managing and mitigating the losses caused by *P. infestans* were estimated around USD 6 billion in 2015 [23,26,27].

*P. infestans*’ life cycle is achieved throughout two pathways. Since this organism is known to be heterothallic, sexual reproduction requires the meeting of two different mating types, namely A1 and A2 [21]. Mating actions lead to the formation of diploid oospores, which establish genetic variations within the populations. Genetic recombination occurring during sexual reproduction is a key phenomenon for the apparition of new resistant or virulent populations [28]. In addition, oospores also constitute survival structures able to persist in soil for relatively long periods of time. Nevertheless, asexual multiplication is most commonly used for dissemination of the disease across the fields [18]. Indeed, along with its mycelial growth, *P. infestans* develops sporangia [24]. Sporangia can either directly germinate to infect plant tissues when temperatures are relatively high (around 20–25 °C) or release motile zoospores produced within them at lower temperatures (between 10 and 15 °C) [26,29]. Zoospores are biflagellate cells that need moisture to swim towards new hosts and participate in additional infection.

At the early stage of infection, spores germinate at the surface of plant tissues by creating appressoria that are able to enter into host cells. It is the biotrophic phase during which the first symptoms appear: a white felting starts progressing on the abaxial side of the leaves [30]. Later on, the pseudofungi switches to the necrotrophic phase and feeds itself by absorbing plant cellular content [30]. This initiates necrosis during advanced stages of the infection. It ends up blocking photosynthesis and slowing down tuberization. The combination of both these trophic stages is called hemibiotrophy [29]. Globally, the pathogen survives thus by the persistence of its mycelium but disseminates thanks to the density of its spores [19]. Infected plants and tubers are therefore the primary source of inoculum. This is why discarding infected tissues remains the first prophylactic action useful to avoid potato late blight outbreaks.

While certain lineages (such as US-1, US-8 [31], or EU-13 [32]) have gained legendary status over the years because of their persistence across different parts of the world [33], new strains of *P. infestans* are rapidly emerging [34]. These appear to be more virulent, develop resistances to previously effective substances (e.g., phenylamides as metalaxyl) [35,36,37] or show reduced sensitivity towards others (fluazinam) [38]. They also reproduce faster and spread more rapidly across fields than before [39]. The emergence of these new pathovars is making the fight against potato late blight disease more relevant and urgent than ever. Taking that into consideration, innovative ways for the management of both old and new strains must be encouraged.

Current global food production heavily relies on intensive agriculture practices along with extensive use of fungicides [40]. The efficacy of these synthetic substances starts to fail because pathogens populations are developing strategies to overcome inhibition properties and became resistant throughout the years [41,42]. In addition, out of over 4 million tons of pesticide produced in 2019 (all chemical families considered), it is estimated that only 0.1% effectively reached the intended target [43]. Consequently, the majority of these chemicals end up in soils, water bodies, or into the atmosphere, contributing to pollution, altering species distribution, and causing the destruction of ecosystems [44]. Moreover, the residues of synthetic pesticides also pose significant risks to human and animal health because they accumulate in tissues and have been associated with various health issues such as cancer, mutagenicity, hepatotoxicity, neurotoxicity, nephrotoxicity, and infertility on both livestock and wild animals [45].

In response to these challenges, there is an urgent need to implement more sustainable and environmentally friendly agricultural practices. Cultural, harvesting and storage methods act as the first lines of action for integrated pest management (IPM) by limiting the dissemination and survival of pathogens [46]. In the case of potato, while numerous cultivars exist, only a limited number of them are grown on a large scale and are valorized by the industry. As it currently stands, the market leaders have been selected based on other criteria such as the yields, the organoleptic properties, and the size and shape of tubers [47]. This has made their growing hardly possible without chemical control [48]. Yet, varietal selection also plays a significant role in disease management [49]. Many studies have demonstrated the effectiveness of resistant varieties exhibiting reduced or even no symptoms of either foliage or tuber late blight [50,51,52,53]. Besides this, among alternative tools, natural molecules including plants metabolites are emerging. Their use in the frame of IPM recently introduced the notion of biocontrol, recently promoted by European legislation [54].

The definition of biocontrol is given as “any agent—originated from nature—used for the management of crop pests”. Unlike common sense would sometimes describe it, biocontrol not only includes the use of micro and macroorganisms but also semiochemicals (pheromones and kairomones) and natural substances coming from plants, animals, or of mineral origins [55,56,57,58,59,60,61,62,63,64].

At this point, many research papers have testified the efficacy of specialized microorganism metabolites against *P. infestans* [65,66,67,68,69,70]. Similarly, several kinds of plant secondary metabolites (PSM) have been reported as well [71,72] such as flavonoids, tannins, coumarins, sterols and alkaloids, but also different kinds of glycosides [73,74,75,76,77,78,79]. On the other hand, researchers also focus on volatile organic compounds specifically found in essential oils (EOs) in order to harness plant’s arsenal while overcoming the constraints.

To the best of our knowledge, nothing reported in the literature provides a clear overview of what has been accomplished on the agent of potato late blight so far. In this paper, we aim to establish a clear overview of the up-to-date works related to the alternative management of *P. infestans* through EOs. Before reaching that point, we will briefly define and classify the metabolites found in essential oils and expose their fields of applications. We will also touch upon certain mechanisms of action on the cellular structures of *Phytophthora* since it has been poorly presented until now [56]. Eventually, we will expose some of the difficulties encountered while working with such volatile compounds and the techniques existing for biopesticides development.

This review attempts to provide a better understanding about the means and reasons EOs could be used to fight phytopathogens such as *P. infestans*.

## 2. Essential Oils as Alternative Management against *P. infestans*

### 2.1. Essential Oils Description and Fields of Application

Essential oils are described as complex hydrophobic substances resulting from plants’ secondary metabolism [80]. They are conventionally extracted through hydrodistillation, steam distillation, or cold pressure [81,82,83]. They mostly encompass a wide diversity of volatile organic compounds (VOCs) normally produced and utilized by plants as means of defense as well as intra- and interspecific communication. More specifically, these metabolites serve purposes as attracting pollinators, repelling herbivores, combating phytopathogens, and ensuring plant immunity [84].

Over the past few decades, essential oils have garnered broad interest in various industrial and research fields, including food, cosmetic, pharmaceutical, agronomic, and medical [85,86]. Their chemical composition provides them with a broad spectrum of biological properties, such as preservative, flavoring, or antioxidant agents [87,88]. Moreover, extensive studies have already attested their diverse properties as insecticides, herbicides, fungicides, antibacterial and antiviral agents, particularly useful in the frame of crop protection [89,90]. Their natural origin, high biodegradability, and generally low toxicity make them promising candidates for the development of new biopesticides for agronomical purposes [91]. Despite promising advantages and the tremendous number of studies conducted in this sense, essential oils have encountered difficulties in becoming established on the market. In fact, biopesticides represent barely 5% of global pesticides sold annually, among which the large majority is based on microorganisms [92]. This puts plant-based products way behind and represent thus an opportunity to be seized.

**Figure 1:**
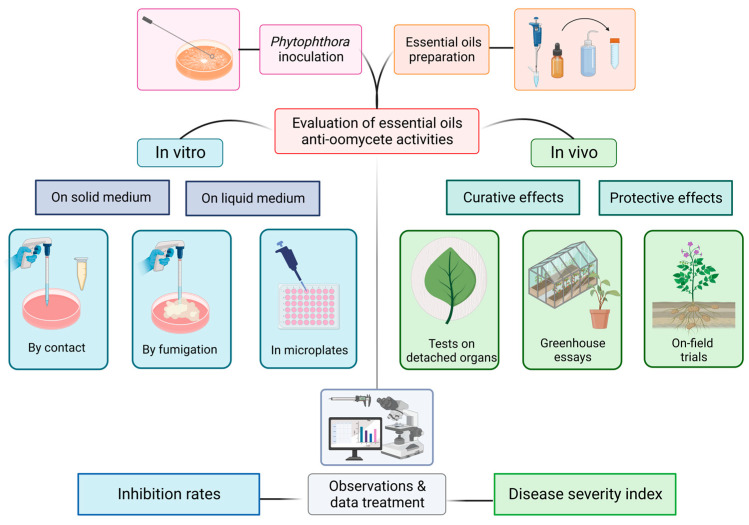
Illustration of the different experimental designs listed in the literature for testing essential oil activities on the development of *Phytophthora* spp.

Among their areas of applications, EOs are listed as control agents of pathogenic microorganisms. Figure 1 illustrates under a technical point of view the distinct protocols on which anti-oomycete activities can be evaluated. We then present in Table 1 what has been conducted against *Phytophthora* spp. with a special focus on *P. infestans*. Simultaneously, we briefly detail the precise experimental design and the associated results obtained. This will engage further discussion about the challenges and the pertinence of the methodologies employed.

### 2.2. Assessing Anti-Oomycete Activities of Natural Substances at Different Laboratory Scales

The biological activity of natural substances against phytopathogens can be evaluated at different levels. To begin, in vitro assays are extensively cited in the literature as a result of their convenience for studying the characteristics of microorganisms. This occurs principally on Petri dishes or in microplates in solid and liquid media, respectively. However, it is important to keep in mind that these assays only represent an initial step in biopesticide development. Indeed, many studies confine their experiments away from real conditions. As a consequence, it prevents the apprehension of the microorganism behavior in its natural environment in response to the tested substances.

Conversely, as can be observed in Table 1, documentation on in vivo assays is more limited. This is mainly due to the higher complexity of the experimental setup. Studying potato late blight under real conditions involves the control of the *S. tuberosum*–*P. infestans* pathosystem. This clearly requires significant resources and time compared to in vitro assays that can be carried out much faster. Nevertheless, the pathosystem must be implemented in order to understand the actual interactions existing between the pathogen and its host. It provides evidence for the anti-oomycete effects of active substances under conditions that are as close as possible to real agroecosystems. In addition, in vivo tests can be conducted at various scopes.

Firstly, detached leaf assays (DLA) offer an initial approximation of the plant’s reaction to an infection. As mentioned earlier, leaves are typically the first organ colonized by the late blight agent. Once the spores reach the leaf surface, they initiate the germination process and start developing mycelium. This marks the progression of the disease [127]. It results in the apparition of a white felting typically observed and measurable at the early stage of late blight. Nonetheless, maintaining detached leaves intact has its limits. Chlorophyll degradation, drying, and bacterial contamination are a few examples that hinder the long-term conductance of those ex situ experiments. Consequently, DLA may not always correlate with in situ tests [128].

Secondly, in vivo experiments can be performed on whole plants in controlled greenhouse conditions. This allows disease monitoring on the natural host with optimal control of the pathosystem parameters: temperature, humidity, and photoperiod. They can all significantly influence the development of *P. infestans* [129]. Another great deal of interest when switching on living material is the varietal choice. Indeed, tolerant and susceptible cultivars do not react the same when facing pathogens [102]. Resistance mechanisms (R-genes particularly) largely influence the development of the disease [130,131]. Furthermore, distinct *Phytophthora* strains or isolates belonging to the same species but sampled from different areas would not react the same manner to the same substances [100,132] nor express equivalent virulence on plant host [93]. Although belonging to the same species and causing the same pathology, those populations still exhibit various stages of virulence, resistance, and rapidity to accomplish their life cycle [133]. Inevitably, this also contributes to the variability of the results obtained.

Thirdly, studies conducted on field consider numerous parameters that impact not only the physiology of the pathogen but also the response of the crop [65]. These include soil physicochemical properties, meteorology, climate, agronomic practices (e.g., fertilizers and pesticides history, plowing), the presence or absence of other micro-/macroorganisms, and, of course, the interactions they hold with the pathosystem. Lastly, on-field trials require a long period of time, large areas, and above all a comprehensive data collection to ensure accurate interpretation of results. Unlike experiments carried out in the laboratory (i.e., on Petri dish, in microplates, detached leaves or even on whole plants in greenhouse), environmental factors cannot be controlled here. Favorable conditions at one point may become unfavorable later, adding complexity to the experiments. In conclusion, the sequential changes of experimental scales (in vitro, ex vivo, in vivo) are time- and resource-consuming for the operator, whereas successful laboratory results do not always lead to promising situation on the field [134].

### 2.3. Insights of Essential Oils Activity on P. infestans

In order to correctly discuss protocols carried out in different conditions by different researchers, adapted comparison values must be chosen. The activity of a substance in vitro is commonly described as an inhibitory threshold such as IC/EC_50_ or IC/EC_90_, or via a precise % of inhibition at a specific concentration—most often either on mycelium growth or on spore production or germination. In vivo, the reduction in disease severity index (DSI/DSR) is used as indicator of either curative or protective properties.

If relevant, these orders of magnitude will be mentioned to facilitate comparisons.

Initially, large in vitro screenings usually serve as preliminary indicators for selecting highly bioactive compounds with great potential. For instance, Quintanilla et al. and De Clerck et al. conducted studies on extensive variety of EOs against *P. infestans* [95,110]. Afterwards, certain EOs are selected for further investigation. As referenced in Table 1, it is already worth emphasizing the frequency at which some botanical families such as Lamiaceae (notably with thyme, peppermint, and oregano), Lauraceae (mainly cinnamon) Myrtaceae (clove and eucalyptus) and Rutaceae are presented in the literature. Interestingly, the majority of these taxa are part of the most manufactured EOs around the world [135] and benefit in some cases from large biomass wastes that need to be valorized [82].

During in vitro experiments, mycelium growth occurs as the main parameters monitored to evaluate anti-oomycete power. In that context, EOs can be tested either in liquid phase (i.e., dissolved in the culture medium with or without an organic solvent and/or a surfactant)—one qualifies this “by contact”—or in vapor phase during what is called “fumigation”. From there, it has been demonstrated several times that most of the time, the vapor phase acts to a much greater extent against *Phytophthora* spp. than by contact [94,96,100,109,136]. Several protocols were implemented in order to assess mycelium inhibition on Petri dish and fumigations were by far, the most effective [97]. For instance, complete inhibition was achieved with as little as 0.3 µg/mL air for both oregano and thyme oils by fumigation whereas it required up to 6.4 µg/mL in liquid medium to achieve the same inhibition by contact [94]. Several other EOs follow that trend. It is, however, not the case for all. Fennel EOs has an EC_50_ ≈ 8 µg/mL in liquid phase whereas its vapor phase could simply not cause any inhibition at all, even at the highest concentrations [100]. This is probably due to the lower vapor pressure of the bioactive compounds that prevents them from acting on mycelium when not in contact with it. Eventually, exposure time is a critical parameter to assess properly the efficacy of fumigations treatments since volatile compounds take some time to go from liquid to gaseous state [136].

In addition, in vitro experiments can also deal with sporangia and spores’ production and their ability to achieve germination. Usually, EOs give better results on reproductive structures (i.e., sporangia and spores) than on vegetative ones (mycelium). De facto, effective concentration relative to inhibition of both spores’ production and germination are commonly lower to those relative to mycelium development. This happened on many *Phytophthora* spp. illustrated here: *P. infestans* [94], *P. capsici* [100], *P. nicotianae* [118] and even on *P. parasitica* [99].

Beyond distinct effects on various structures of the pathogens, EOs with very similar composition can conversely generate very contrasting results. Three Rutaceae oils were compared, and bergamot’s much better activity compared with orange and lemon was hypothesized to be caused by their slight distinction of minor components [120]. Similarly, three *Thymus* species were compared from a chemical and biological perspective. Despite their closely related phylogeny, the EOs extracted from these three plant species differed significantly in their profiles of secondary metabolites. Consequently, the essential oils exhibited gradual fumigant effects on *P. infestans*, at 60%, 80%, and 100% inhibition, respectively, for *T. convolutus*, *T. pectinatus*, and *T. vulgaris* [96].

Subsequently, oils can further be applied onto whole plants; disease progression monitored under controlled conditions. Interestingly, a study conducted on two potato varieties of different susceptibility towards late blight revealed that one of the EOs tested (hyssop) not only prevented disease progression, but also appeared to enhance plant growth [95]. Clearly, EOs impact the development of the pathogen as much as they modulate plant physiology. Therefore, negative plant response such as phytotoxicity must be considered [137]. Taking this into account, Quintanilla et al. established a qualitative evaluation of phytotoxicity and expressed the potential of the tested EOs in regard to both crop protection and phytotoxic effects [95].

Ultimately, favorable biological properties are sometimes revealed when trials are extended in the field. For instance, orange oil was not particularly active against mycelium growth on Petri dish but reduced late blight progression in greenhouse experiments by up to 80% at a concentration of 5 mL/L [121]. Jointly, rosemary offered the best protection on potato plants against late blight, whereas thyme and clove were, in contrast, the best inhibitors on Petri dishes and microplates [93]. Hence, when EO effectiveness is assessed both in vitro and in vivo, trends in results may occasionally diverge. This makes the selection of promising chemical biocontrol agent even more delicate. Moreover, when trials are pushed far enough in time, treated plants end up as infected as the control ones [102]. This demonstrates the limit of protection conferred by EOs when simply applied without appropriate formulation [83].

Conjointly, EOs are regularly more effective for preventive applications (i.e., applied before inoculation) than for curative uses (i.e., applied after inoculation) [121,122,123]. These two modes of application clearly target two opposing but complementary stages of the disease. Prevention aims to block spore germination, while the purpose of curative treatments is to slow down or stop mycelium progression throughout the leaves. As mentioned earlier, spores seem more sensitive, i.e., inhibited at lower concentrations than mycelium. This correlates with the better performance of EOs as a preventive treatment rather than curative.

Finally, innovative formulations regularly tested in vitro, significantly enhanced the inhibition over time compared with the oil tested alone [103,104,107,108].

**Figure 2:**
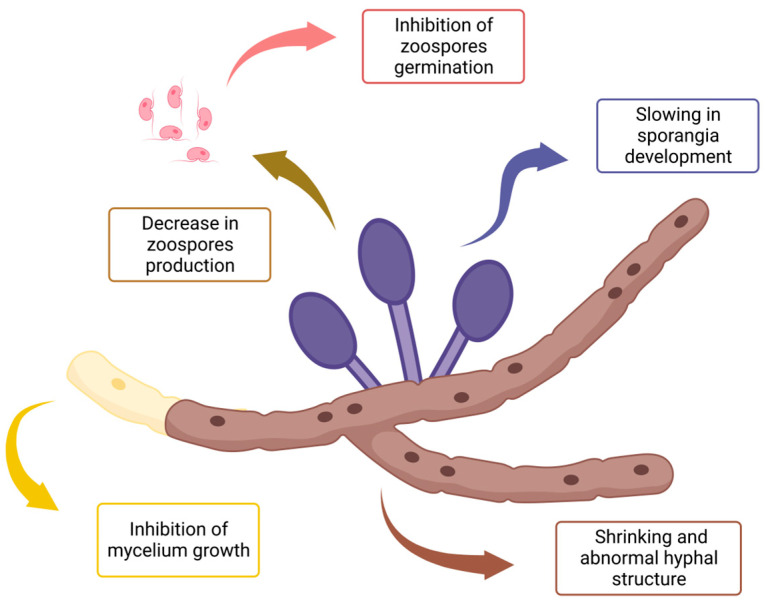
Summary of EO impacts on the development of *Phytophthora infestans* mycelial structures and reproductive forms, all possible experimental devices taken into account.

The effects of EOs throughout all listed experimental designs are summarized in Figure 2. As mentioned previously, anti-oomycete activities can occur towards hyphae structure and growth as well as the development, production, and germination of reproductive forms.

## 3. Investigating Mechanisms of Action of Essential Oil Components

### 3.1. Chemical Composition and Variability of Essential Oils

Essential oils are complex mixtures of volatile organic compounds (VOCs) generated by plants secondary metabolism [138]. They mostly—but not exclusively—gather two major types of compounds that can be classified based on the metabolic pathway they come from: terpenoids and phenylpropanoids [139].

Terpenic compounds result from the condensation of several isoprene unit (IU—C_5_H_8_) [140]. Monoterpenes (2 IU—C_10_) together with sesquiterpenes (3 IU—C_15_) frequently account for the majority of essential oil composition [141], although diterpene (4 IU—C_20_) and triterpene (6 IU—C_30_) also exist [142]. Theoretically, “terpenes” strictly refers to linear or cyclic unsaturated hydrocarbons, whereas “terpenoids” carry various oxygenated functions which give alcohols, ethers, ketones, aldehydes, or esters [91].

On the other hand, phenylpropanoids are synthetized from the phenylalanine amino acid notably through the shikimate pathway [138,143]. The chemical structure involves a benzene ring to which other organic functions can be attached [144]. Phenylpropanoids occur less frequently than terpenes and are specific to microorganisms and plants [141].

Comparing complex mixtures is not an easy task. Indeed, some species of plants can exhibit completely different chemical compositions and, consequently, distinct biological activities of their oil. In fact, plant secondary metabolism can be influenced by both endogenous and exogenous factors [145]. Endogenous factors refer to the plant genetic, anatomic and physiological features from which the biomass will be extracted [146]. On the other hand, exogenous or abiotic factors encompass environmental parameters into which plants develop. This includes soil properties, altitude, meteorological as well as climatic conditions (e.g., temperature, humidity, light, and photoperiod) [147,148,149,150,151]. Agronomic practices—cultivation methods and fertilization or the physiological stage at which the plant is harvested—are other causes responsible for EO variability [152].

In other words, biotic and abiotic conditions among which plants grow along with the extraction method—modify EO chemical composition in both qualitative (type of metabolites) and quantitative (their proportion) manner [105].

To overcome confusion, the chemotype must be specified. A chemotype refers to a chemically distinct profile of secondary metabolites derived from the same plant species [83,153]. Small genetic or epigenetic differences can significantly alter the chemotype of a plant and, consequently, the composition of its essential oil, even if the plant’s morphology appears unchanged. Thyme serves as a notable example, with at least seven different chemotypes identified within the same species [154]. Beyond the notion of chemotype, the major/leading compounds also serve to describe EOs—in an approximative way but with reasonable accuracy [155].

So far, we have always considered essential oils as substances in their own right. Nevertheless, in order to correctly apprehend their mechanisms of action, chemical profile must be known. Gas chromatographic analysis coupled with mass spectrometry (GC-MS) has become an essential tool to determine precise compositions of EOs [139,156]. Regrettably, all studies do not systematically provide a complete GC-MS analysis of the studied oils. Though, in order to better understand underlying molecular patterns, it seems essential to be aware at least of the main compounds involved.

For this purpose, we reported in Table 2 —when indicated—the major compound(s) along with the plant from which the EOs were extracted. These VOCs are classified according to their metabolic pathway of origin, together with the chemical family they belong to. They will further be discussed as promising molecules for late blight disease control. Other plant extracts containing high proportions of the listed molecules are likely to show appropriate anti-oomycete potentials as well.

### 3.2. Cellular Impacts of Essential Oil Components on Oomycetes

**Figure 3:**
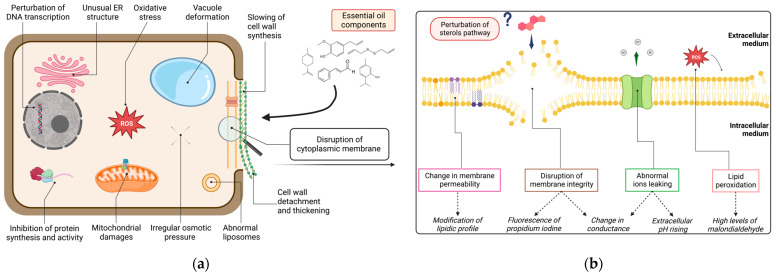
Possible mechanisms of all reported EO and EOCs on *Phytophthora* cellular sites: (**a**) general impacts on DNA transcription, protein synthesis and activity, osmotic pressure, endoplasmic reticulum, liposomes, vacuoles and mitochondria structures, cell wall structure, and cytoplasmic membrane integrity. (**b**) Zoom on deduced mechanisms (in box) towards plasmalemma: membrane disruption, change in permeability, lipidic peroxidation and ions leaking with the associated parameters assessing these damages (in italic) and hypothetical perturbation of sterol pathway.

In order to precisely apprehend the mechanisms whereby essential oil components (EOCs) act on pathogens, attention must be drawn to a molecular scope. Figure 3a provides a graphical representation of the possible cellular sites of action of EOs bioactive molecules on oomycetes. Since the cell membrane was revealed to be a hot spot for this topic, Figure 3b zoomed in to examine precise phenomena occurring on that specific target, along with the main associated parameters observed to assess them.

As presented above, several modes of action have been highlighted on different cellular sites of *Phytophthora*. Still, EOs activities are much more understood on bacteria, fungi or weeds than on oomycetes [54,91,137,159,160]. Since the literature lacks comprehension on *P. infestans* specifically, we extended the scope to *Phytophthora* spp. and more generally to oomycetes.

To begin, EOC must access the right cellular location before carrying out any biological activity. Unlike plants whose tissues are generally protected by a cuticular wax [161], oomycete mycelium and spores present a relatively simple histology. It enables a faster translocation of molecules directly towards the cells.

Limited information is provided about the impact of EOCs on the oomycete cell wall, the first cellular barrier to cross. However, Soylu et al. mentioned cell wall detachment and thickening, which was certainly attributed to an inappropriate biosynthesis of polysaccharides [94]. Some monoterpenes (D-limonene, α-terpinene, and p-cymene) were demonstrated to disturb cell wall structure on bacteria [162] and fungi [163]. Due to the distinctions with oomycete cell wall, we can only hypothesize similar action of terpenic compounds, without guaranteeing it. It is worth highlighting that chemical nature and functional group position characterizing EOC clearly influence their efficacy [99,101]. This might explain for example, the difference of biological activity between isomers such as thymol and carvacrol. These two phenolic compounds differ in the position of the hydroxyl group around the aromatic ring. They might interact with hydrophobic sugars and therefore disrupt the cell wall with different levels of affinity.

When confronted with any signs of cytotoxicity, *Phytophthora* mobilizes detoxification tools. Among those commonly found against conventional fungicides, there are efflux pumps, cell wall bonding [164] or enzymatic complexes such as cytochromes P450 [165]. These defense mechanisms either remove the toxic compound from the cytosol or transform it into a non-toxic one. None of them were proven to act specifically towards EOC. Yet, some plant metabolites—notably, thymol or carvacrol—have been described as efficient inhibitors of efflux pump, but on other microorganisms [166,167]. Similarly, if the efflux of VOCs is prevented by one of them, global efficacy would certainly be enhanced.

Generally, VOCs biocidal activities towards microorganisms are mostly related to the lipophilic nature, low molecular weight and high vapor pressure [84,160]. In that context, cell membranes are key targets for terpenoids and phenylpropanoids. These molecules easily interact with phospholipids, fatty acid and sterols, perturbating general membrane integrity [42]. Several biological parameters were reported on *Phytophthora* plasmalemma and will be listed as evidence of its disruption.

First, membrane electrical conductivity reflects on electrolytes balance. Changes in membrane permeability leads to ions leakage [168] which results in abnormal conductance [169,170]. This has been observed on *P. capsici* and *P. nicotianae* facing turmeric oil [115], eugenol (a leading compounds of clove oils—among others) [171], *C. indicum* and *Z. armatum* oils (almost exclusively composed of mono- and sesquiterpene) [118,123] as well as diallyl disulfide (main component of garlic oil) [119]. Additionally, a decrease in the pH was measured after treatment with eugenol, manifesting abnormal ions flow (in this case protons) across the membrane [171]. This feature is relatively common with VOCs bearing a hydroxyl group (carvacrol and eugenol for instance). This chemical function increases the hydrophilic nature of the molecule which slightly enhances solubility in aqueous medium. It also gives the ability to easily exchange protons [103].

Second, malondialdehyde (MDA) is a common product of reactive oxygen species (ROS) reacting with polyunsaturated fatty acids [172]. In the same way, cellular content of *Phytophthora* spp. in MDA was measured after facing several treatments with diallyl disulfide, eugenol, curcumol or D-limonene, for instance. When mycelial inhibition occurred, cells showed MDA rates proportional to substances concentrations [115,118,124,171]. Although it probably indicates oxidative stress around the membrane, MDA does not reveal the oxidation of one specific molecule. Yet, a precise target needs to be identified in order to correctly apprehend EOC oxidative abilities on the oomycete plasmalemma.

Generally, terpenic hydrocarbons seem less likely to disrupt bacteria cytoplasmic membrane—notably through oxidations—compared with oxygenated terpenoids [173]. The same assumption could be extended to oomycetes. This was considered by the presence of geranial, geraniol, or nerol when D-limonene was the major compounds (<90%) in the oils of several citrus [120]. Linalol was also proved to affect lipids metabolism on another oomycete (*Saprolegnia ferax*) [174]. It modified the permeability of both cytoplasmic and mitochondrial membranes which impacted cellular flow and respiration processes. Similar results were reported on other fungal phytopathogens: thymol was thought to be responsible for lipid peroxidation and even to interfere with ergosterol biosynthesis [175,176,177].

Thirdly, propidium iodine (PI) is a fluorescent probe that crosses damaged plasma membrane and binds to DNA [178]. It is used to detect dying cells through membrane degradations. Correspondingly to the previous listed markers (conductivity, pH and MDA levels), PI fluorescence increased when *Phytophthora* hyphae were confronted with some oil treatments [118,124]. While this observation effectively attests membrane damage, precise lipidic profile should indicate molecular alteration of specific membrane components.

Once into the intracellular medium, EOCs also interfere with the cytoplasmic content. Osmotic pressure can be revealed by the detection of excessive levels of glycerol in fungi-like organisms [179]. To be precise, intracellular glycerol levels increased in a turmeric oil dose-dependent manner [115]. Interestingly, vacuoles swelled and became unusually larger [94,124], whereas liposomes were also detected abnormal or completely absent compared to non-treated cells [94,115,123,124]. The endoplasmic reticulum (ER) continuity also became unusual facing methyleugenol [150].

To sum up, essential oil lipophilic nature is proposed to primarily degrade the cytoplasmic membrane. Nevertheless, EOC manifestly perturb organelles as well. As known, they all are delimited by a membrane although their composition in lipids and proteins clearly varies [180]. Since they are part of the endo-membrane system connected to the plasmalemma, we propose to extend the general mechanisms of EOC to all organelles delimited by such a lipidic structure. This forms what could look like a cellular continuum highly likely to represent a prime target for lipophilic compounds i.e., terpenes and phenylpropanoids.

Moreover, some other mechanisms of common terpenes have been reported but consistently on bacteria or fungi rather than on oomycetes [42]. Still, it is worth noting that linalol affects some protein complex involved in the respiratory chain while mitochondrial dysfunction by D-limonene was also reported [123,181]. In addition, terpinen-4-ol was in turn proved to disturb DNA transcription and protein synthesis [182]. Oxygenated VOCs bearing polar groups reportedly participate in the inhibition of some major enzyme complex by bonding easily to their active site through the formation of hydrogen bonds [183]. Once again, regarding the fundamental cytologic differences between bacteria, fungi versus pseudo-fungi, no strict conclusions may here be drawn. Yet, these assumptions open up certain attempts to understand. Lastly, citral inhibited the expression of certain effector genes and could decrease virulence of *P. capsici* towards its host [184]. This raises a whole new field of comprehension on a transcriptomic level about pathogen–host interactions modulated by EOC.

### 3.3. VOCs Interactions Modulate Biological Activities

The potential of EO heavily relies on the combined activities of the multiple compounds they are made out of. In fact, some cases reported that isolated compounds show better activity than the whole essential oil at equivalent concentration (e.g., diallyl disulfide compared to garlic oil) [119]. In contrast, the whole mixture regularly works better (e.g., curcumol and β-elemene compared to turmeric oil [115]; D-limonene and linalol compared to the oil of winged prickly [123]; thymol compared to thyme oil [167]). Consequently, EOC are proved to work either in synergy, with additive effects or as antagonists [90,185].

## 4. Overcoming Essential Oils Challenges for Biopesticides Development

Natural products are gaining interests due to their ability to be easily degraded and thus less persistent in the environment, unlike most synthetic pesticides [186]. Above all, they have shown multiples times biological potential to fight crop disease. However, these benefits also lead to some challenges to overcome. Indeed, low persistency in the environment means a shorter time lapse during which the molecules display biological actions. In fact, natural compounds are usually unstable outside of the cell compartments they originally come from [85]. High volatility and instability facing oxygen, light, or heat, contribute to EOC lack of persistence [187]. Furthermore, the hydrophobic nature of essential oils makes it physically difficult to develop biopesticides sprayable on the fields because those must be in aqueous solution to be practically used. Therefore, adapted formulations must be established to overcome these challenges before considering the breakthrough of plant-based phytosanitary products.

In that field, research has led to much progress since many different and innovative formulations have already been published. Their purpose is to enhance the slow release of essential oils onto their target, reduce volatility, increase stability, and improve water solubility. All these parameters are primordial to ensure spreading and penetration of active substances throughout the living tissues. In addition, chemical degradations (occurring through oxidation and isomerization, for instance) must also be prevented to conserve for as long as possible the initial properties [188]. Formulation helps release progressively bioactive molecules towards the target at the most appropriate time [189].

In that context, micro and nanoemulsions are very documented strategies used to formulate EOs. They both consist of homogeneous isotropic colloidal systems where droplets of EO are dispersed in an aqueous solution with the help of a surfactant and occasionally co-surfactants [190]. The distinction between these two types of emulsions lies in the size of dispersed oil droplets related to the free Gibbs energy of the system [191]. The main issue to overcome is finding the appropriate balance between the biologically active compounds and the most adapted surfactant agent. In fact, the activity of EO emulsions has been proven to vary similarly to the physico-chemical parameters of the emulsions, notably according to droplet size, which influences the stability [192,193,194].

On the other hand, encapsulation refers to any physical or chemical techniques allowing to enclose and protect a substance to release it in a controlled way [83]. Nowadays, the most appropriate matrix to encapsulate biopesticides seems to be natural polymers [195,196]. As an example, cyclodextrins are so-called “molecular cages” and intensively studied for their properties enabling the confinement of VOCs in 3D-structures [197,198].

Finally, many other appropriate ways exist to overcome EO challenges and develop such biopesticides—both in liquid and solid state. In any case, it must be carefully chosen according to the application, the agronomic context, and proper targets in order for them to effectively work [199].

## 5. Discussion

**Figure 4:**
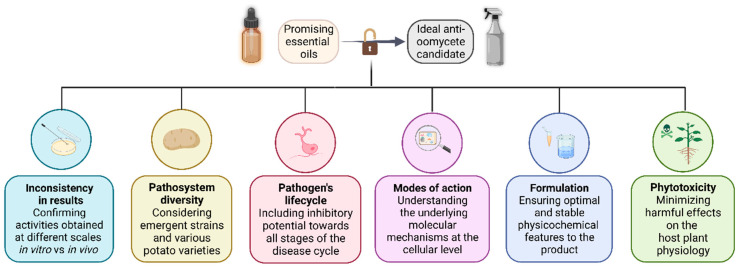
Summary chart of the concerns addressed during the development of an essential oil-based fungicide candidate.

We have presented some reasons explaining the limited number of EO-based products registered for crop protection, despite numerous studies demonstrating the potential of those secondary metabolites. On Figure 4, we summarized the concerns during the development of a biofungicide candidate based on essential oils. Nevertheless, further steps do exist—but are beyond this paper scope —and need to be taken before the actual launch on the market of such products.

Pathogen behaviors fluctuate according to conditions and laboratory settings whereas plant physiology can respond in many different ways to the infection. On the other hand, EO efficacy also depends on the chosen surfactants because these systematically modify the biodisponibility of active ingredients [200]. The versatility of essential oils paired with the complexity of *S. tuberosum*—*P. infestans* pathosystem reflects on the high diversity of protocols that can be tested. Therefore, it is the researchers’ responsibility to report the limits of the results provided in the laboratory, in greenhouses or in the field. De facto, no breakthrough of news molecules may be promoted as long the efficacy has not been tested at each experimental and practical scale.

While most of the time, they clearly interfere with mycelium growth, EOC also slows the development of sporangia and spores [100]. Hence, the impact towards different tissues is complementary because cell lysis of vegetative apparatus impedes reproductive organs development [113]. Evidently, if zoospores cannot be produced nor released, it drastically reduces the rate of dissemination of the pathogen [201] and thus the progression of the disease. While some compounds do affect both vegetative and reproductive structures of *Phytophthora* [113], others are efficient only on either one of them, leaving the other relatively intact [171]. To optimize efficacy, research and development focus on substances able to inhibit both vegetative and reproductive forms. If it acts at different stages of the life cycle and through multiple mechanisms of action, the biocidal power is more likely to successfully express. Nevertheless, plant infection is the one to prioritize and reduce above all stages of the lifecycle.

In order to prevent as much phytotoxicity as possible and guarantee valuable crop yields [202], there is a need to develop an effective method against *P. infestans* that will not (or little) interfere with *S. tuberosum* physiology [159]. Knowing that the cell membrane appeared similar to the major site of action of EOCs, we suggest focusing on one of its specific components.

We mentioned earlier certain particularities of oomycetes cytology. Another interesting trait is the inability for some species to synthetize their own sterols [203], a characteristic called auxotrophy. Sterols designate a class of compounds derived from triterpenoids that ensures—among other roles—membrane fluidity and integrity [204]. Auxotrophic organisms need to acquire these metabolites by absorption from the cellular content of host plants because they are unable to synthetize de novo [205]. Sterols are common to all living organisms but differ in terms of origin and chemical structures (phytosterols in plants [206] and ergosterol in fungi [207]). In the case of *Phytophthora*, certain constituents of essential oils might interfere with sterols absorption because *Phytophthora* does not (or very little) modify them before integration onto its plasma membrane [208]. In conclusion, to achieve good action of a novel substance, the aim is to ensure that its mechanism relies towards a cellular site, or a specific metabolite only involved in the homeostasis of the pathogen but not (or as little as possible) in the one of non-target organisms: plant-host, insects, or soil microbiome [209].

Apprehending global effects of substances is crucial to guarantee low environmental toxicity and qualify them as "low-risk" [59] or “generally recognized as safe” (GRAS) [210]. A growing number of synthetic molecules are being forbidden because of dangerous impacts on human, animal or ecosystem health and need to be replaced [211,212,213].

Furthermore, EO mixture of active and sometimes multi-target molecules are particularly useful on resistant pathogens [167]. As described earlier, disparate cellular sites can be disturbed simultaneously by more than on molecules. This puts a lower selection pressure and decreases chances for the apparition of resistant populations [214].

Lastly, the lesser discussed benefits of EO in the frame of plant protection are known as eliciting and priming effects [215,216]. They consist of preparing crops to better fight pathogens or apprehend physiologic stresses by inducing plant defenses [217]. The effects of EO on plant immunity just began to be investigated.

Nowadays, global food system production is being undermined notably by climate change [218], loss of soil fertility and novel resistances to pesticides. Besides this, aromatic and perfume plants—from which most of common EOs are extracted—usually require significant amounts of fertilizers or phytochemicals to reach acceptable biomass yields [219]. Therefore, growing those plants with conventional and intensive practices to promote afterwards a sustainable agriculture with plant-based biopesticides—would seem like nonsense. Moreover, primary resources (water, arable lands, and energy—among others) are monopolized in some countries for the production of EO. In some cases, it jeopardizes the survival of local populations.

In brief, the increased demand for EO throughout the years has resulted in severe environmental and social impacts in some countries of the world. For these reasons, biomass origin and production methods must absolutely be regulated.

Several other factors contribute to the poor adoption rate of EO as biopesticides: strict legislation—particularly in the EU compared to the USA, China, or India—[220] low and sometimes inconsistent persistence of biological activities due to chemical variability and difficulties to standardize quality and quantity of the production [221]. Lastly, low yields of extraction impede affordable prices of EOs, which makes it difficult for them to substitute synthetic pesticides—generally much more affordable [222].

Up to now, only mint and orange EOs (with L-carvone [223] and D-limonene as main constituents, respectively) have been registered in some countries of Europe, as potato anti-sprouting agents [224]. Other than that, no EO-based treatment exists on the market against potato diseases. However, modern monitoring techniques establish potato late blight diagnostic and plan at best necessary phytochemicals treatments [225,226].

Incontestably, the actual farming world cannot yet work correctly and ensure current and future needs of food production without conventional pesticides [227]. Nonetheless, partial substitution of synthetic molecules with plant-based products [228], together with appropriate methods could ensure a more optimal and sustainable crop protection.

## 6. Conclusions

- Context on potato and associated diseases: *Solanum tuberosum* is one of the most important crops in terms of human consumption and *Phytophthora infestans*—an oomycete causing potato late blight—represents its main threat.
- Challenges for late blight control: Synthetics pesticides are harmful to human health, the environment, and biodiversity; thus, biocontrol tools, in particular, natural molecules extracted from plants, such as essential oils, are gaining interest.
- Current research status: Numerous in vitro studies demonstrated the efficacy of essential oils, but in vivo trials are still lacking.
- Inconsistencies in the results: Essential oils tested against *P. infestans* are not unanimous in their effectiveness and do not systematically present same potential at different experimental and practical scales.
- Incomplete understanding of mechanisms of action: Essential oil components primarily target cell walls and membranes but also other cellular structures, which must be further explored.
- High diversity of VOCs composition: Investigation on essential oil major compounds may allow better comprehension of the global mechanisms of action.
- Main challenges for EO-based biopesticides: Finding substances that specifically disturb *Phytophthora* cellular machinery without impacting the host plant (phytotoxicity) nor other living organisms (ecotoxicity).
- Need for optimal formulation: EO requires appropriate physico-chemical methods to ensure stability, target-specific delivery, and long-term activity.
- Take-home message: Essential oils definitely present high anti-oomycete potential to cure diseases such as late blight caused by *Phytophthora infestans*; however, cellular sites of action must be better understood, and appropriate formulations developed to obtain effective biopesticides.

## Figures and Tables

**Table 1:** Overview of literature references on essential oils classified according to the botanical origin (family, genus, and species) tested against *Phytophthora* spp. with the experimental design and associated results.

Essential Oil Origin			Tested			References
Botanical Family	Vernacular Name	Plant Species	In Vitro Experiments (EO Concentration—Results Obtained)	In Vivo Experiments (EO Concentration—Results Obtained)	On *Phytophthora*	
Lamiaceae	Thyme	*Thymus* *vulgaris*	Sporangial germination on microplate (ED_50_ ≈ 0.3 µL/mL) Mycelium growth inhibition on Petri dish (80% MGI from 0.41 µL/mL)	Detached leave assays (DSI at 0% from 3.33 µL/mL) Greenhouse assays on potato plants (DSR = 80% at 3.33 µL/mL)	*infestans*	[93]
			Fumigation test against mycelium growth (total inhibition from 0.3 µg/mL air) Contact test against mycelium growth (total inhibition from 6.4 µg/mL) Contact effect on sporangia production (Absence of sporangia from 1.6 µg/mL)	-		[94]
			Mycelium growth inhibition on Petri dish (90% inhibition CTC with 4 µL/plate after 22 days)	Greenhouse experiments on 2 potato cultivars (1:500 *v*/*v*—reduction of 30 and 40% of DSI CTC)		[95]
			Fumigation test (100% inhibition at 1 µL/Petri dish and LC_50_ = 0.467 µL/mL air)	-		[96]
			Fumigation test on mycelium growth (Mycelium area ≈ −85% CTC after 19 days)	-		[97]
			Sporangia development on microplate (IC_50_ = 99.41 mg/L)	-		[98]
			Mycelium growth inhibition on agar (Inhibition of 55% CTC at 100 ppm)	-		[24]
			Mycelium growth inhibition on Petri dish (95% inhibition at 144 and ED_50_ ≈ 70 mg/L) Sporangia development (Completely blocked from 72 mg/L) Zoospores production and germination (100% inhibited from 72 mg/mL)	-	*parasitica*	[99]
			Mycelium growth inhibition (EC_50_ ≈ 0.14 µg/mL by contact and EC_50_ ≈ 0.11 µg/mL by fumigation) Sporangia and zoospores production ((EC_50_ ≈ 0.0475 µg/mL) Sporangia and zoospores germination (EC_50_ ≈ 0.095 µg/mL)	-	*capsici*	[100]
		*Thymus* *satureioides*	Mycelium growth inhibition on agar (Inhibition of 80% CTC at 100 ppm)	-	*infestans*	[24]
		*Thymus* *convoltus*	Fumigation test on Petri dish (60% inhibition at 4 µL/Petri after 7 days LC_50_ ND)	-		[96]
		*Thymus* *pectipatus*	Fumigation test on Petri dish (100% inhibition CTC at 2 µL/Petri after 7 days, LC_50_ = 0.452 µL/mL air)	-		[96]
		*Thymus* *capitus*	Antifungal activity on mycelium growth (IC_50_ = 107 µL/L)	-		[101]
		*Thymus* *algeriensis*	Antifungal activity on mycelium growth (ND)	-		[101]
		*Thymus* *schimperi*	-	On-field assays on 2 potato cultivars with ≠ levels of resistance (DSI equal to controls from 46 DAP)		[102]
		*Thymus* *serpyllum*	Test by contact in Petri dish with EO encapsulated in lignin nanoparticles (LNP) (EC_50_ = 120 µg/mL for EO alone and EC_50_ = 88 µg/mL for EO-LNP)	Greenhouse tests on black pine plantlets (−20% mortality CTC with EO and no mortality with EO-LNP after 10 days)	*cactorum*	[103]
	Oregano	*Origanum* *vulgare*	Fumigation test against mycelium growth (total inhibition from 0.3 µg/mL air) Contact test against mycelium growth (total inhibition from 6.4 µg/mL) Contact effect on sporangia production (Absence of sporangia from 0.8 µg/mL)	-	*infestans*	[94]
			Fumigation test on mycelium growth (Mycelium area ≈ −50% CTC after 19 days)	-	*infestans*	[97]
			Mycelium growth inhibition on Petri dish (60% inhibition CTC with 4 µL/plate after 22 days)	-		[95]
			Mycelium growth inhibition on agar (Inhibition of 90% CTC at 100 ppm)	Potato plants in growth chamber on (25% disease suppression CTC at 0.2%)		[24]
		*Origanum* *compactum*	Sporangia development on microplate (IC_50_ = 96.5 mg/L)	-		[98]
		*Origanum* *syriacum*	Mycelium growth inhibition (EC_50_ ≈ 0.07 µg/mL by contact and EC_50_ ≈ 0.09 µg/mL by fumigation) Sporangia and zoospores production (EC_50_ ≈ 0.0475 µg/mL) and germination (EC_50_ ≈ 0.095 µg/mL)	-	*capsici*	[100]
		*Origanum* *majorana*	Fumigation test on mycelium growth (Mycelium area ≈ −35% CTC after 19 days)	-	*infestans*	[97]
			Mycelium growth inhibition on agar (Inhibition EO < formulation EO + PANAM)	-		[104]
	Savory	*Satureja* *montana*	Sporangia development on microplate (IC_50_ = 74.65 mg/L)	-		[98]
	Rosemary	*Rosmarinus* *officinalis*	Sporangial germination on microplate (ED_50_ ≈ 0.6 µL/mL) Mycelium growth inhibition on Petri dish (80% inhibition from 1.66 µL/mL)	Detached potato leaves assays (−30% DSI CTC from 1.66 µL/mL) Greenhouse assays on potato plants (DSR = 90% from 3.33 µL/mL)		[93]
			Fumigation test on mycelium growth (Mycelium area ≈ −15% CTC after 19 days)	-		[97]
			Fumigation test against mycelium growth (total inhibition from 1.2 µg/mL air) Contact test against mycelium growth (total inhibition from 12.8 µg/mL) Contact effect on sporangia production (Absence of sporangia from 6.4 µg/mL)	-		[94]
			-	On-field assays with 2 potato cultivars presenting ≠ levels of resistance (DSI equal to the control from 46 DAP)	*infestans*	[102]
			Mycelium growth inhibition on Petri dish (EC_50_ ≈ 172 µL/L)	-	*nicotianae*	[105]
	Sage	*Salvia* *officinalis*	Fumigation test on mycelium growth Mycelium area ≈ −30% CTC after 19 days)	-	*infestans*	[97]
			Mycelium growth inhibition (EC_50_ ≈ 4.86 µg/mL by contact and EC_50_ ≈ 1.28 µg/mL by fumigation)	-	*capsici*	[100]
	Basil	*Ocinum* *basilicum*	Fumigation test on mycelium growth (Mycelium area ≈ −50% CTC after 19 days)	-	*infestans*	[97]
			Mycelium growth inhibition (ED_50_ ≈ 120 mg/L)	-	*parasitica*	[99]
			Mycelium growth inhibition on Petri dish (Total inhibition around 400 ppm for all three strains and EC_50_ ≈ 135, 200 and 191 ppm, respectively)	Assays in greenhouse on whole plants of pepper, cucumber and melon (DSI reduced by50, 36 and 44% CTC after 50 mL at 100 ppm applied on the roots of inoculated plants)	*capsici* *dreshleris* *melonis*	[106]
	Massep	*Ocinum* *gratissimum*	Mycelium growth inhibition on Petri dish (Total inhibition up to 10 days at 300 µL/L with pure EO and at 250 µL/L with nano-emulsion)	Tests on artificially infected tomato fruits (Disease reduction of 47% and 100% with 900 µL/L of pure EO and nano-emulsion for preventive tests and of 100% with 900 µL/L of both treatments for curative)	*infestans*	[107]
			Mycelium growth inhibition on Petri dish (Total inhibition from 6250 µg/mL after 14 days)	-		[74]
	Pepper Mint	*Mentha* *piperita*	Mycelium growth inhibition on Petri dish (65% inhibition CTC with 4 µL/plate after 22 days)	Greenhouse experiments on 2 potato cultivars (1:500 *v*/*v*—reduction of 10 and 25% of DSI CTC over 22 days)		[95]
			Fumigation test on mycelium growth (Mycelium area ≈ −85% CTC after 19 days)	-	*infestans*	[97]
			Mycelium growth inhibition by fumigation (Total inhibition with 100 µL/Petri dish)	-		[108]
			Mycelium growth inhibition by contact (Total inhibition at 1 µL/mL for all spp.) Mycelium growth inhibition by fumigation (Total inhibition at 25 µL/L air for all spp.)	-	*capsici* *melonis* *nicotianae* *cinnamoni* *citrophthora*	[109]
	Green mint	*Mentha* *spicata*	Sporangia development on microplate (IC_50_ = 130.56 mg/L)	-	*infestans*	[98]
			Mycelium growth inhibition by fumigation (Total inhibition with 100 µL/Petri dish)	-		[108]
		*Mentha* *pulegium*	Sporangial germination on microplate (Total inhibition after 120 h at 1000 ppm)	-		[110]
	Lemon balm	*Melissa* *officinalis*	Mycelium growth inhibition on Petri dish (70% inhibition CTC 4 µL/plate at day 22)	-		[95]
	Lavender	*Lavendula* *officinalis*	Fumigation test against mycelium growth (Total inhibition from 1.6 µg/mL air) Contact test against mycelium growth (Total inhibition from 25.6 µg/mL) Contact effect on sporangia production (Absence of sporangia from 6.4 µg/mL)	-		[94]
			Mycelium growth inhibition on agar (100 ppm—inhibition of 20% CTC)	-		[24]
			Mycelium growth inhibition by contact (Total inhibition at 5 µL/mL for all spp.) Mycelium growth inhibition by fumigation (Total inhibition at 250 µL/L air for all spp.)	-	*capsici* *melonis* *nicotianae* *cinnamoni* *citrophthora*	[109]
	Patchouli	*Pogostemon* *cablin*	Fumigation test on mycelium growth (Mycelium area ≈ −25% CTC after 19 days)	-	*infestans*	[97]
	Hyssop	*Hyssopus* *officinalis*	Mycelium growth inhibition on Petri dish (45% inhibition CTC with 4 µL/plate after 22 days)	Greenhouse experiments on 2 potato cultivars (1:500 *v*/*v*—reduction of 70 and 85% of DSI CTC)	*infestans*	[95]
	Zataria	*Zataria* *multiflora*	Mycelium growth inhibition on Petri dish in association with chitosan (CS) (EO IC_50_ = 0.039% and EO + CS IC_50_ = 0.011%	Assays on wounded cucumber fruits artificially infected (DSI −20% CTC with EO alone and −75% CTC with EO-CS after 7 days at 4 °C and then 2 days at 24°)	*drechsleri*	[111]
Myrtaceae	Clove	*Syzygium* *aromaticum*	Sporangial germination on microplate (ED_50_ ≈ 4.5 µL/mL) Mycelium growth inhibition on Petri dish (80% MGI from 0.41 µL/mL)	Detached potato leaves assays (DSI −30% CTC from 3.33 µL/mL) Greenhouse assays on potato plants (DSR = 40% at 6.66 µL/mL)	*infestans*	[93]
			Sporangia development on microplate (IC_50_ = 28.42 mg/L)	-		[98]
			Mycelium growth inhibition (Total inhibition from 250 µL/L)	Tests on cocoa pod husk pieces (DSI −70% CTC at 1000 µL/L after 2 weeks)	*megakarya*	[112]
	Eucalyptus	*Eucalyptus* *globulus*	-	On-field assays on 2 potato cultivars with ≠ levels of resistance (DSI −25% on sensitive 53 DAP and DSI −33% on resistant cultivar 60 DAP)	*infestans*	[102]
		*Eucalyptus* *citriodora*	Sporangia development on microplate (IC_50_ = 122.11 mg/L)	-		[98]
		*Eucalyptus* *tereticornis*	Mycelium growth inhibition on Petri dish (Total inhibition from 12,500 µg/mL after 14 days)	-		[74]
	Tea tree	*Melaleuca* *alternifolia*	Mycelium growth inhibition (EC_50_ ≈ 3.59 µg/mL by contact and EC_50_ ≈ 10.07 µg/mL by fumigation)	-	*capsici*	[100]
			Sporangia development on microplate (IC_50_ = 476.37 mg/L)	-	*infestans*	[98]
Lauraceae	Laurel	*Laurus* *nobilis*	Fumigation test against mycelium growth (Total inhibition from 2.0 µg/mL air) Contact test against mycelium growth (Total inhibition from 51.2 µg/mL) Contact effect on sporangia production (Absence of sporangia from 12.8 µg/mL)	-	*infestans*	[94]
	Cinnamon	*Cinnamomum* *cassia*	Sporangial germination on microplate (ED_50_ ≈ 0.5 µL/mL) Mycelium growth inhibition on Petri dish (80% MGI from 1.66 µL/mL)	Detached potato leaves assays (DSI −40% CTC at 6.66 µL/mL) Greenhouse assays on potato plants (DSR = 20% at 6.66 µL/mL)		[93]
			Sporangial germination on microplates (Total inhibition after 120 h at 1000 ppm)	-		[110]
			Mycelium growth inhibition on Petri dish (Total inhibition at 72 and ED_50_ ≈ 40 mg/L) Sporangia and zoospores production (Completely blocked from 144 mg/L) Zoospores germination (Totally inhibited from 72 mg/mL)	-	*parasitica.*	[99]
		*Cinnamomum* *zeylanicum*	Sporangial germination on microplate (Total inhibition after 120 h at 1000 ppm)	-	*infestans*	[110]
			Mycelium growth inhibition on Petri dish in association with chitosan (CS) (EO IC_50_ = 0.039% and EO + CS IC_50_ = 0.011%)	Assays on wounded cucumber fruits (DSI −35% CTC with EO alone and −85% CTC with EO-CS after 7 days at 4 °C and then 2 days at 24°)	*drechsleri*	[111]
			Mycelium growth inhibition (EC_50_ ≈ 0.19 µg/mL by contact and EC_50_ ≈ 0.28 µg/mL by fumigation)	-	*capsici*	[100]
			Mycelium growth inhibition on Petri dish (Total inhibition from 0.625 mg/mL) Zoospores’ germination (Totally inhibited from 0.625 mg/mL) Sporangia production (Totally impeded from 1.25 mg/mL)	Effect on leaf necrosis and sporulation on taro aerial part leaves (Disease symptoms completely inhibited—leaf necrosis diameter = 0—and sporulation entirely blocked from 1.25 mg/mL)	*colocasiae*	[113]
Cupressaceae	Juniper	*Juniperus* *communis*	Sporangial germination on microplate (ED_50_ ≈ 2.3 µL/mL) Mycelium growth inhibition on Petri dish (30% MGI from 3.33 µL/mL)	Detached potato leaves assays (DSI −25% CTC from 3.33 µL/mL) Greenhouse assays on potato plants (DSR around 40% at 3.33 µL/mL)	*infestans*	[93]
Verbenaceae	Common lantana	*Lantana* *camara*	Mycelium growth on Petri dish (40% inhibition CTC at 2 mL/L after 7 days)	-		[114]
Piperaceae	Pepper	*Piper* *nigrum*	Sporangial germination on microplates (ED_50_ ≈ 1.2 µL/mL) Mycelium growth inhibition on Petri dish (30% MGI at 6.66 µL/mL)	Detached leave assays (DSI −30% CTC from 6.66 µL/mL) Greenhouse assays on potato plants (DSI around 50% at 3.33 µL/mL)		[93]
Zingiberaceae	Turmeric	*Curcuma* *longa*	Sporangial germination on microplates (ED_50_ ≈ 2.5 µL/mL) Mycelium growth inhibition on Petri dish (60% MGI from 3.33 µL/mL)	Detached leave assays (DSI −25% CTC from 3.33 µL/mL) Greenhouse assays on potato plants (DSR = 75% from 3.33 µL/mL)		[93]
		*Curcuma* *phaeocaulis*	Mycelium growth inhibition on Petri dish (EC_50_ = 4.9 µg/mL and EC_90_ = 34.3 µg/mL)	-		[115]
			Mycelium growth inhibition on Petri dish (EC_50_ = 0.5 µg/mL and EC_90_ = 7.1 µg/mL) Investigation of activity against sporangial and zoospore production and germination (No sporangial nor zoospore production at 20 µg/mL and spores’ takes 4× more time to achieve germination at 20 µg/mL CTC)	Protective and curative assays on detached cucumber leaves (Control efficacy > 90% CTC for both preventive and curative activities from 100 µg/mL after 72 h of incubation)	*capsici*	[115]
	Ginger	*Zingiber* *officinalis*	Mycelium growth on Petri dish (100% inhibition at 2 mL/L for 7 days)	Greenhouse assays on tomato plants (DSI −80% CTC after 10 weeks)	*infestans*	[114,116]
			Mycelium growth inhibition on Petri dish (Total inhibition from 1250 ppm) Inhibition of sporangia and zoospores (Total inhibition for at 625 ppm)	Assessment of necrosis on taro leaves (Diameter of necrosis ≈0 and no from 1250 ppm sporangia after 72 h) Reduction in symptoms on taro corms (−80% DSI CTC at 625 ppm after 7 days)	*colocasiae*	[117]
Asteraceae	Mexican marigold	*Tagetes* *erecta*	Mycelium growth inhibition on Petri dish (40% inhibition CTC at 2 mL/L after 7 days)	Greenhouse assays on tomato plants (DSI −80% CTC after 10 weeks)	*infestans*	[114,116]
	Indian chrysanthemum	*Chrysanthemum* *indicum*	Mycelium growth inhibition on Petri dish (100% inhibition CTC from 200 µL/L) Spore germination (100% inhibition CTC from 200 µL/L) Fumigation test on mycelium growth (100% inhibition from 100 µL/L)	-	*nicotianae*	[118]
Amaryllidaceae	Garlic	*Allium* *sativum*	-	On-field assays on 2 potato cultivars presenting ≠ levels of resistance (DSI −33% CTC 53 DAP on susceptible −33% CTC up to 60 DAP on resistant)	*infestans*	[102]
			Mycelium growth on Petri dish (100% inhibition at 2 mL/L after 7 days)	Greenhouse assays on tomato plants (DSI −80% CTC after 10 weeks)		[114,116]
			Mycelium growth inhibition on Petri dish in DMSO 2% (EC_50_ ≈ 1 108 µL/L after 4 days)	On-pot experiments on tobacco roots (Disease control effect of 46% by root- irrigation at 1:500 *v*/*v* and of 49% by fumigation at 1:500 *v*/*v*)	*nicotianae*	[119]
Rutaceae	Lemon	*Citrus* *limon*	Mycelium growth inhibition on Petri dish (35% inhibition CTC after 7 days) Inhibition rate of sporulation (10% inhibition CTC at 1:100 *v*/*v* at day 21)	Inhibition of the infection on potato foliar discs after soaking in EO solutions at 3 dilution rates (Average inhibition of 5% CTC)	*infestans*	[120]
	Orange	*Citrus* *sinensis*	Mycelium growth inhibition on Petri dish (No inhibition at all tested concentrations 1.15; 2.5; 5; 7.5 mL/L)	Greenhouse experiments (−80% DSI CTC for protective at 5 mL/L but no curative effect at 7.5 mL/L)		[121]
			Mycelium growth inhibition on Petri dish (50% inhibition CTC after 7 days) Inhibition rate of sporulation (90% inhibition CTC at 1:100 *v*/*v* at day 21)	Inhibition of the infection on potato foliar discs after soaking in EO solutions at 3 dilution rates (Average inhibition of 65% CTC)		[120]
			Mycelium growth inhibition by contact (No inhibition for none of the spp. even at the highest tested concentration of 1 µL/mL)	-	*capsici* *melonis* *nicotianae* *cinnamoni* *citrophthora*	[109]
	Bergamot	*Citrus* *bergamia*	Mycelium growth inhibition on Petri dish (55% inhibition CTC after 7 days) Inhibition rate of sporulation (50% inhibition CTC at 1:100 *v*/*v* at day 21)	Inhibition of the infection on potato foliar discs after soaking in EO solutions at 3 dilution rates (Average inhibition of 40% CTC)	*infestans*	[120]
	Lime	*Citrus* *aurantifolia*	Mycelium growth inhibition on Petri dish (MGI > 95% at 400 ppm after 7 days) Inhibition of sporangium production (−50% sporangia at 250 ppm CTC)	Necrosis inhibition tests on taro foliar discs (At 5000 ppm total necrosis inhibition for preventive and 50% for curative test)	*colocasiae*	[122]
	Bottle brush	*Callistermon* *citrinus*	Mycelium growth inhibition on Petri dish (Total inhibition from 312.5 µg/mL after 14 days)	-	*infestans*	[74]
	Prickly ash	*Zanthoxylum* *armatum*	Mycelium growth inhibition on Petri dish (Total inhibition at 2.5 µL/mL from 48 h) Investigation of activity against sporangial and zoospore production and germination (No sporangial nor zoospore production and germination at 2.0 µg/mL)	Protective and curative tests on pepper fruits (Control efficacy > 90% CTC for protective and 80% for curative at 200 µL/mL after 3 days of incubation)	*capsici*	[123]
		*Zanthoxylum* *xanthoxyloides*	Mycelium growth inhibition on Petri dish (Total inhibition from 350 µL/L)	Tests on cocoa pod husk pieces (DSI −64% CTC at 2000 µL/L after 2 weeks)	*megakarya*	[112]
	/	*Tetradium* *glabrifolium*	Mycelium growth inhibition on Petri dish (Total inhibition at 20 mg/L up to 96 h) Activity against spores’ production (No spores produced at all at 20 mg/L) Inhibition of spores’ germination (3× more time to germinate at 10 mg/L CTC)	Activity test on detached pepper leaves (Efficacy ≈ 100% CTC at 500 mg/L for both protective and curative after 96 h) Activity test on pepper fruits (Efficacy ≈ 100% CTC at 500 mg/L for both protective and curative after 96 h)	*capsici*	[124]
Poaceae	Lemon Grass	*Cymbopogon* *nardus*	Mycelium growth inhibition (EC_50_ ≈ 0.44 µg/mL by contact and EC_50_ ≈ 0.25 µg/mL by fumigation)	-	*capsici*	[100]
		*Cymbopogon* *flexuosus*	Sporangial germination on microplates (Total inhibition for 120 h at 1000 ppm)	-	*infestans*	[110]
		*Cymbopogon* *citratus*	Mycelium growth inhibition on Petri dish (Total inhibition around 72.5 ppm for all three strains and EC_50_ ≈135, 200 and 191 ppm, respectively)	On-plants assays in greenhouse (DSI reduced by 30, 70 and 35% CTC after 50 mL at 100 ppm applied on the roots of inoculated plants)	*capsici* *dreschsleri* *melonis*	[106]
			Mycelium growth inhibition on Petri dish (No growth at 6250 µg/mL after 14 days)	-	*infestans*	[74]
			Mycelium growth inhibition on Petri dish (Total inhibition at 244 and ED_50_ ≈ 60 mg/L) Sporangia and zoospores production (Totally hampered at 144 mg/L) Zoospores germination (Totally inhibited from 72 mg/mL)	-	*parasitica*	[99]
	Palmarosa	*Cymbopogon* *Martini*	Mycelium growth inhibition (EC_50_ ≈ 0.10 µg/mL by contact and EC_50_ ≈ 0.15 µg/mL by fumigation) Sporangia and zoospores production ((EC_50_ ≈ 0.04 µg/mL) and germination (EC_50_ ≈ 0.08 µg/mL)	-	*capsici*	[100]
Euphorbiaceae	Croton	*Croton* *macrostachyrus*	-	On-field assays on 2 potato cultivars presenting ≠ levels of resistance (DSI −25% CTC 53 DAP on susceptible and −15% CTC 60 DAP on resistant)	*infestans*	[102]
Apiaceae	Caraway	*Carum* *carvi*	Mycelium growth inhibition on Petri dish (4 µL/plate—no inhibition at all CTC after 22 days)	Greenhouse experiments on 2 potatoes cultivars (1:500 *v*/*v*—reduction of 15 and 35% of DSI CTC over 22 days)		[95]
			Mycelium growth inhibition (ND)	-		[125]
			Fumigation test against mycelium growth (total inhibition from 0.4 µg/mL air) Contact test against mycelium growth (total inhibition from 6.4 µg/mL) Contact effect on sporangia production (Absence of sporangia from 3.2 µg/mL)	-		[94]
	Cumin	*Cuminum* *cymimum*	Mycelium growth inhibition on Petri dish (Total inhibition at 216 and ED_50_ ≈60 mg/L) Sporangia and zoospores production (80% inhibited from 144 mg/L) Zoospores germination (Totally inhibited from 144 mg/mL)	-	*parasitica*	[99]
			Mycelium growth inhibition on Petri dish (10% inhibition CTC with 4 µL/plate after 22 days)	Greenhouse experiments on 2 potatoes cultivars (DSR of 20 and 30% CTC after 22 days at 1:500 *v*/*v*)	*infestans*	[95]
	Fennel	*Foeniculum* *vulgare*	Mycelium growth inhibition (EC_50_ ≈ 8.10 µg/mL by contact but no inhibition at all by fumigation)	-	*capsici*	[100]
	Dill	*Anethum* *graveolens*	Mycelium growth inhibition on Petri dish (ND)			[126]
Cannabaceae	Hop	*Humulus* *lupulus*	Mycelium growth on twelve-well plates (IC_50_ > 1000 mg/L) Spores’ germination on microplates (IC_50_ > 5000 mg/L)	-	*infestans*	[76]
Geraniaceae	/	*Pelagornium graveolens*	Mycelium growth inhibition (ED_50_ ≈ 140 mg/L)	-	*parasitica*	[99]
	Geranium	*Geranium* spp. (ND)	Mycelium growth inhibition by contact (Total inhibition at 1 µL/mL for all spp.) Mycelium growth inhibition by fumigation (Total inhibition at 100 µL/L air for all spp.)	-	*capsici* *melonis* *nicotianae* *cinnamoni* *citrophthora*	[109]

MGI: mycelium growth inhibition; DSI: disease severity index; DSR: disease severity reduction; IC_50_: median inhibitory concentration; ED_50_: median effective dose; LC_50_: median lethal concentration; CTC: compared to control (non-treated); DAP: days after planting; ND: no data.

**Table 2:** Major constituents identified in promising essential oils against *Phytophthora* spp., classified according to the metabolic pathways and chemical class they belong to.

Chemical Class	Metabolite	CAS Number	Found as Major Component in the Essential Oils of	Reference
Terpenoids				
Hydrocarbons	D-limonene	5989−27-5	*Citrus limon*	[120]
			*Citrus bergamia*	
			*Citrus sinensis*	
			*Zanthoxylum armatum*	[123]
			*Tetradium glabrifolium*	[124]
			*Citrus aurantifolia*	[122]
	α-pinene	80-56-8	*Rosmarinus officinalis*	[102,105]
			*Cistus ladanifer*	[157]
	α-terpinene	99-86-5	*Origanum majorana*	[104]
	α-selinene	473-13-2	*Chrysanthemum indicum*	[118]
	α-humulene	6753-98-6	*Humulus lupulus*	[76]
	γ-terpinene	99-85-4	*Thymus vulgaris*	[96]
			*Ocimum gratissimum*	[107]
	*€*-β-caryophyllene	87-44-5	*Humulus lupulus*	[76]
	α-phellandrene	99-83-2	*Anethum graveolens*	[126]
	p-cymene	99-87-6	*Thymus pectipatus*	[96]
			*Origanum marjorana*	[104]
	β-ocimene	3779-61-1	*Ocimum basilicum*	[106]
	β-elemene	33880-83-0	*Tetradium glabrifolium*	[124]
	δ-cadimène	483-76-1	*Chrysanthemum indicum*	[118]
Phenolic compounds	Carvacrol	499-75-2	*Thymus vulgaris*	[94,96]
			*Thymus schimperi*	[102]
			*Origanum vulgaris*	[94]
			*Origanum compactum*	[98]
			*Satureja montana*	[98]
			*Thymus capitatus*	[101]
			*Thymus serpyllum*	[103]
	Thymol	89-83-8	*Thymus vulgaris*	[98,99,100,101]
			*Thymus pectinatus*	[96]
			*Ocimum gratissimum*	[107]
			*Zataria multiflora*	[111]
Alcohol	Borneol	507-70-0	*Thymus satureioides*	[132]
			*Ocinum basilicum*	[106]
			*Rosmarinus officinalis*	[94]
	Linalol	78-70-6	*Ocinum basilicum*	[99]
			*Zanthoxylum armatum*	[123]
	Terpinen-4-ol	562-74-3	*Melaleuca alternifolia*	[98]
	Citronellol	106-22-9	*Pelargonium graveolens*	[99]
			*Zanthoxylum xanthoxyloides*	[112]
	Geraniol	106-24-1	*Citrus aurantifolia*	[122]
	Curcumol	4871-97-0	*Curcuma zedoaria*	[115]
Ether	Eucalyptol	470-82-6	*Eucalyptus globulus*	[102]
			*Laurus nobilis*	[94]
			*Thymus convoltus*	[96]
			*Origanum majorana*	[104]
			*Curcuma zedoaria*	[115]
	Dill ether	74410-10-9	*Anethum graveolens*	[126]
Ketone	L-carvone	6485-40-1	*Mentha spicata*	[98,108]
	D-carvone	2244-16-8	*Anethum graveolens*	[126]
			*Carum carvi*	[125]
	L-menthone	89-78-1	*Mentha piperita*	[108]
	Camphor	76-22-2	*Lavendula officinalis*	[94]
			*Thymus convolutus*	[96]
Aldehyde	Citronellal	106-23-0	*Eucalyptus citriodara*	[98,105]
	Neral—Citral B	106-26-3	*Cymbopogon citratus*	[106]
	Geranial—Citral A	5392-40-5	*Cymbopogon citratus*	[99,106,121]
			*Citrus sinensis*	[121,122]
			*Zingiber officinale*	[117]
Ester	Bornyl acetate	76-49-3	*Citrus aurantifolia*	[122]
Phenylpropanoids				
Ether	Eugenol	97-53-0	*Syzygium aromaticum*	[93,98,112]
			*Syringa oblata*	[158]
	Methyleugenol	93-15-2	*Asarum heterotropoides*	[159]
	Anethole	104-46-1	*Foeniculum vulgare*	[94]
Aldehyde	Cuminaldehyde	122-03-2	*Cuminum cyminum*	[99]
	Cinnamaldehyde	14371-10-9	*Cinnamomum cassia*	[99]
			*Cinnamomum zeylanicum*	[111,111]
Other metabolic pathway				
Sulfur compounds	Diallyl disulfide	2179-57-9	*Allium sativum*	[119]

## Data Availability

Not applicable.

## References

[1] Devaux A., Goffart J.-P., Petsakos A., Kromann P., Gatto M., Okello J., Suarez V., Hareau G., Campos H., Ortiz O. (2020). Global Food Security, Contributions from Sustainable Potato Agri-Food Systems. The Potato Crop: Its Agricultural, Nutritional and Social Contribution to Humankind.

[2] Haverkort A.J., Struik P.C. (2015). Yield Levels of Potato Crops: Recent Achievements and Future Prospects. Field Crops Res..

[3] Raymundo R., Asseng S., Robertson R., Petsakos A., Hoogenboom G., Quiroz R., Hareau G., Wolf J. (2018). Climate Change Impact on Global Potato Production. Eur. J. Agron..

[4] Djaman K., Koudahe K., Koubodana H.D., Saibou A., Essah S. (2022). Tillage Practices in Potato (*Solanum tuberosum* L.) Production: A Review. Am. J. Potato Res..

[5] Haverkort A.J. (1990). Ecology of Potato Cropping Systems in Relation to Latitude and Altitude. Agric. Syst..

[6] Dupuis J.H., Liu Q. (2019). Potato Starch: A Review of Physicochemical, Functional and Nutritional Properties. Am. J. Potato Res..

[7] Navarre D.A., Goyer A., Shakya R., Singh J., Kaur L. (2009). Chapter 14—Nutritional Value of Potatoes: Vitamin, Phytonutrient, and Mineral Content. Advances in Potato Chemistry and Technology.

[8] Godfray H.C.J., Beddington J.R., Crute I.R., Haddad L., Lawrence D., Muir J.F., Pretty J., Robinson S., Thomas S.M., Toulmin C. (2010). Food Security: The Challenge of Feeding 9 Billion People. Science.

[9] Cooke D.E.L., Drenth A., Duncan J.M., Wagels G., Brasier C.M. (2000). A Molecular Phylogeny of Phytophthora and Related Oomycetes. Fungal Genet. Biol..

[10] Kroon L.P.N.M., Brouwer H., de Cock A.W.A.M., Govers F. (2012). The Genus Phytophthora Anno 2012. Phytopathology.

[11] Sogin M.L., Silberman J.D. (1998). Evolution of the Protists and Protistan Parasites from the Perspective of Molecular Systematics. Int. J. Parasitol..

[12] Crous P.W., Rossman A.Y., Aime M.C., Allen W.C., Burgess T., Groenewald J.Z., Castlebury L.A. (2021). Names of Phytopathogenic Fungi: A Practical Guide. Phytopathology.

[13] Carlile M.J., Gow N.A.R., Gadd G.M. (1995). The Success of the Hypha and Mycelium. The Growing Fungus.

[14] Werner S., Steiner U., Becher R., Kortekamp A., Zyprian E., Deising H.B. (2002). Chitin Synthesis during in Planta Growth and Asexual Propagation of the Cellulosic Oomycete and Obligate Biotrophic Grapevine Pathogen Plasmopara Viticola. FEMS Microbiol. Lett..

[15] Chérif M., Benhamou N., Belanger R. (2011). Occurrence of Cellulose and Chitin in the Hyphal Walls of Pythium Ultimum: A Comparative Study with Other Plant Pathogenic Fungi. Can. J. Microbiol..

[16] Ivanov A.A., Ukladov E.O., Golubeva T.S. (2021). *Phytophthora infestans*: An Overview of Methods and Attempts to Combat Late Blight. J. Fungi.

[17] Goss E.M., Tabima J.F., Cooke D.E.L., Restrepo S., Fry W.E., Forbes G.A., Fieland V.J., Cardenas M., Grünwald N.J. (2014). The Irish Potato Famine Pathogen *Phytophthora infestans* Originated in Central Mexico Rather than the Andes. Proc. Natl. Acad. Sci. USA.

[18] Grünwald N.J., Flier W.G. (2005). The Biology of *Phytophthora infestans* at Its Center of Orgin. Annu. Rev. Phytopathol..

[19] Ristaino J.B. (2002). Tracking Historic Migrations of the Irish Potato Famine Pathogen, *Phytophthora infestans*. Microbes Infect..

[20] Schumann G.L., D’Arcy C.J. (2017). CHAPTER 1: The Irish Potato Famine: The Birth of Plant Pathology. Hungry Planet: Stories of Plant Diseases.

[21] Nowicki M., Foolad M.R., Nowakowska M., Kozik E.U. (2012). Potato and Tomato Late Blight Caused by *Phytophthora infestans*: An Overview of Pathology and Resistance Breeding. Plant Dis..

[22] Fawke S., Doumane M., Schornack S. (2015). Oomycete Interactions with Plants: Infection Strategies and Resistance Principles. Microbiol. Mol. Biol. Rev..

[23] Fry W.E., Birch P.R.J., Judelson H.S., Grünwald N.J., Danies G., Everts K.L., Gevens A.J., Gugino B.K., Johnson D.A., Johnson S.B. (2015). Five Reasons to Consider *Phytophthora infestans* a Reemerging Pathogen. Phytopathology.

[24] Olanya M., Anwar M., He Z., Larkin R., Honeycutt C. (2016). Survival Potential of *Phytophthora infestans* Sporangia in Relation to Environmental Factors and Late Blight Occurrence. J. Plant Prot. Res..

[25] Hagman J.E., Mårtensson A., Grandin U. (2009). Cultivation Practices and Potato Cultivars Suitable for Organic Potato Production. Potato Res..

[26] Fry W. (2008). *Phytophthora infestans*: The Plant (and R Gene) Destroyer. Mol. Plant Pathol..

[27] Cui H., Ren X., Yun L., Hou Q., Feng F., Liu H. (2018). Simple and Inexpensive Long-Term Preservation Methods for *Phytophthora infestans*. J. Microbiol. Methods.

[28] Gavino P.D., Smart C.D., Sandrock R.W., Miller J.S., Hamm P.B., Lee T.Y., Davis R.M., Fry W.E. (2000). Implications of Sexual Reproduction for *Phytophthora infestans* in the United States: Generation of an Aggressive Lineage. Plant Dis..

[29] Leesutthiphonchai W., Vu A.L., Ah-Fong A.M.V., Judelson H.S. (2018). How Does *Phytophthora infestans* Evade Control Efforts? Modern Insight Into the Late Blight Disease. Phytopathology.

[30] Kamoun S., Furzer O., Jones J.D.G., Judelson H.S., Ali G.S., Dalio R.J.D., Roy S.G., Schena L., Zambounis A., Panabières F. (2015). The Top 10 Oomycete Pathogens in Molecular Plant Pathology. Mol. Plant Pathol..

[31] Fry W.E., McGrath M.T., Seaman A., Zitter T.A., McLeod A., Danies G., Small I.M., Myers K., Everts K., Gevens A.J. (2013). The 2009 Late Blight Pandemic in the Eastern United States—Causes and Results. Plant Dis..

[32] Cooke D.E.L., Cano L.M., Raffaele S., Bain R.A., Cooke L.R., Etherington G.J., Deahl K.L., Farrer R.A., Gilroy E.M., Goss E.M. (2012). Genome Analyses of an Aggressive and Invasive Lineage of the Irish Potato Famine Pathogen. PLoS Pathog..

[33] Fry W.E. (2016). *Phytophthora infestans*: New Tools (and Old Ones) Lead to New Understanding and Precision Management. Annu. Rev. Phytopathol..

[34] Beninal L., Bouznad Z., Corbière R., Belkhiter S., Mabon R., Taoutaou A., Keddad A., Runno-Paurson E., Andrivon D. (2022). Distribution of Major Clonal Lineages EU_13_A2, EU_2_A1, and EU_23_A1 of *Phytophthora infestans* Associated with Potato Late Blight across Crop Seasons and Regions in Algeria. Plant Pathol..

[35] Shattock R.C. (2002). *Phytophthora infestans*: Populations, Pathogenicity and Phenylamides. Pest Manag. Sci..

[36] Gisi U., Cohen Y. (1996). Resistance to Phenylamide Fungicides: A Case Study with *Phytophthora infestans* Involving Mating Type and Race Structure. Annu. Rev. Phytopathol..

[37] Elansky S., Pobedinskaya M.A., Kokaeva L., Statsyuk N., Dyakov Y.T. (2015). *Phytophthora infestans* Populations from the European Part of Russia: Genotypic Structure and Metalaxyl Resistance. J. Plant Pathol..

[38] Troussieux S., Gilgen A., Souche J.-L. (2022). A New Biocontrol Tool to Fight Potato Late Blight Based on Willaertia Magna C2c Maky Lysate. Plants.

[39] Puidet B., Mabon R., Guibert M., Kiiker R., Soonvald L., Le V.H., Eikemo H., Dewaegeneire P., Saubeau G., Chatot C. (2022). Examining Phenotypic Traits Contributing to the Spread in Northern European Potato Crops of EU_41_A2, a New Clonal Lineage of *Phytophthora infestans*. Phytopathology.

[40] Sharma A., Kumar V., Shahzad B., Tanveer M., Sidhu G.P.S., Handa N., Kohli S.K., Yadav P., Bali A.S., Parihar R.D. (2019). Worldwide Pesticide Usage and Its Impacts on Ecosystem. SN Appl. Sci..

[41] Miller S.A., Ferreira J.P., LeJeune J.T. (2022). Antimicrobial Use and Resistance in Plant Agriculture: A One Health Perspective. Agriculture.

[42] Álvarez-Martínez F.J., Barrajón-Catalán E., Herranz-López M., Micol V. (2021). Antibacterial Plant Compounds, Extracts and Essential Oils: An Updated Review on Their Effects and Putative Mechanisms of Action. Phytomedicine.

[43] Devrnja N., Milutinović M., Savić J. (2022). When Scent Becomes a Weapon—Plant Essential Oils as Potent Bioinsecticides. Sustainability.

[44] Tilman D., Cassman K.G., Matson P.A., Naylor R., Polasky S. (2002). Agricultural Sustainability and Intensive Production Practices. Nature.

[45] Choudhary S., Yamini N.R., Yadav S.K., Kamboj M., Sharma A. (2018). A Review: Pesticide Residue: Cause of Many Animal Health Problems. J. Entomol. Zool. Stud..

[46] Tsedaley B. (2014). Late Blight of Potato (*Phytophthora infestans*) Biology, Economic Importance and Its Management Approaches. J. Biol. Agric. Healthc..

[47] Forbes G.A. (2012). Using Host Resistance to Manage Potato Late Blight with Particular Reference to Developing Countries. Potato Res..

[48] Runno-Paurson E., Williams I.H., Metspalu L., Kaart T., Mänd M. (2013). Current Potato Varieties Are Too Susceptible to Late Blight to Be Grown without Chemical Control under North-East European Conditions. Acta Agric. Scand. Sect. B—Soil Plant Sci..

[49] Haverkort A.J., Boonekamp P.M., Hutten R., Jacobsen E., Lotz L.A.P., Kessel G.J.T., Visser R.G.F., van der Vossen E.A.G. (2008). Societal Costs of Late Blight in Potato and Prospects of Durable Resistance Through Cisgenic Modification. Potato Res..

[50] Kefelegn H., Chala A., Kassa B., Pananjay G., Tiwari K. (2012). Evaluation of Different Potato Variety and Fungicide Combinations for the Management of Potato Late Blight (*Phytophthora infestans*) in Southern Ethiopia. Int. J. Life Sci..

[51] Mekonen S., Tadesse T. (2018). Effect of Varieties and Fungicides on Potato Late Blight (*Phytophthora infestans*, (Mont.) de Bary) Management. Agrotechnology.

[52] Tähtjärv T., Tsahkna A., Tamm S. (2013). Comparison of Late Blight Resistance and Yield of Potato Varieties. Proc. Latv. Acad. Sci. Sect. B Nat. Exact Appl. Sci..

[53] Gedlu D., Hailu N., Kefelegn H. (2023). Integrated Management of Potato Late Blight (*Phytophthora infestans* (Mont) de Bary) through Resistant Varieties and Fungicides in North Shewa, Ethiopia. J. Plant Pathol..

[54] Raveau R., Fontaine J., Lounès-Hadj Sahraoui A. (2020). Essential Oils as Potential Alternative Biocontrol Products against Plant Pathogens and Weeds: A Review. Foods.

[55] (2023). What Is Biocontrol.

[56] Lahlali R., Ezrari S., Radouane N., Kenfaoui J., Esmaeel Q., El Hamss H., Belabess Z., Barka E.A. (2022). Biological Control of Plant Pathogens: A Global Perspective. Microorganisms.

[57] Eilenberg J., Hajek A., Lomer C. (2001). Suggestions for Unifying the Terminology in Biological Control. BioControl.

[58] Seiber J.N., Coats J., Duke S.O., Gross A.D. (2014). Biopesticides: State of the Art and Future Opportunities. J. Agric. Food Chem..

[59] Amichot M., Joly P., Martin-Laurent F., Siaussat D., Lavoir A.-V. (2018). Biocontrol, New Questions for Ecotoxicology?. Environ. Sci. Pollut. Res..

[60] Thomas M.B., Willis A.J. (1998). Biocontrol—Risky but Necessary?. Trends Ecol. Evol..

[61] Raymaekers K., Ponet L., Holtappels D., Berckmans B., Cammue B.P.A. (2020). Screening for Novel Biocontrol Agents Applicable in Plant Disease Management—A Review. Biol. Control.

[62] Reddy P.P., Reddy P.P. (2016). Biological Control of Plant Pathogens. Sustainable Crop Protection under Protected Cultivation.

[63] Hashemi M., Tabet D., Sandroni M., Benavent-Celma C., Seematti J., Andersen C.B., Grenville-Briggs L.J. (2022). The Hunt for Sustainable Biocontrol of Oomycete Plant Pathogens, a Case Study of *Phytophthora infestans*. Fungal Biol. Rev..

[64] Stenberg J.A., Sundh I., Becher P.G., Björkman C., Dubey M., Egan P.A., Friberg H., Gil J.F., Jensen D.F., Jonsson M. (2021). When Is It Biological Control? A Framework of Definitions, Mechanisms, and Classifications. J. Pest Sci..

[65] Caulier S., Gillis A., Colau G., Licciardi F., Liépin M., Desoignies N., Modrie P., Legrève A., Mahillon J., Bragard C. (2018). Versatile Antagonistic Activities of Soil-Borne *Bacillus* Spp. and *Pseudomonas* Spp. against *Phytophthora infestans* and Other Potato Pathogens. Front. Microbiol..

[66] Najdabbasi N., Mirmajlessi S.M., Dewitte K., Ameye M., Mänd M., Audenaert K., Landschoot S., Haesaert G. (2021). Green Leaf Volatile Confers Management of Late Blight Disease: A Green Vaccination in Potato. J. Fungi.

[67] Gfeller A., Fuchsmann P., De Vrieze M., Gindro K., Weisskopf L. (2022). Bacterial Volatiles Known to Inhibit *Phytophthora infestans* Are Emitted on Potato Leaves by Pseudomonas Strains. Microorganisms.

[68] Alfiky A., L’Haridon F., Abou-Mansour E., Weisskopf L. (2022). Disease Inhibiting Effect of Strain Bacillus Subtilis EG21 and Its Metabolites against Potato Pathogens *Phytophthora infestans* and *Rhizoctonia solani*. Phytopathology.

[69] Tran H., Ficke A., Asiimwe T., Höfte M., Raaijmakers J.M. (2007). Role of the Cyclic Lipopeptide Massetolide A in Biological Control of *Phytophthora infestans* and in Colonization of Tomato Plants by Pseudomonas Fluorescens. New Phytol..

[70] De Vrieze M., Germanier F., Vuille N., Weisskopf L. (2018). Combining Different Potato-Associated Pseudomonas Strains for Improved Biocontrol of *Phytophthora infestans*. Front. Microbiol..

[71] Aqil F., Zahin M., Ahmad I., Owais M., Khan M.S.A., Bansal S.S., Farooq S., Ahmad I., Owais M., Shahid M., Aqil F. (2010). Antifungal Activity of Medicinal Plant Extracts and Phytocompounds: A Review. Combating Fungal Infections: Problems and Remedy.

[72] Kumar J., Ramlal A., Mallick D., Mishra V. (2021). An Overview of Some Biopesticides and Their Importance in Plant Protection for Commercial Acceptance. Plants.

[73] Abdelgaleil S., Zoghroban A., El-Bakry A., Shehata M. (2019). Insecticidal and Antifungal Activities of Crude Extracts and Pure Compounds from Rhizomes of *Curcuma longa* L. (Zingiberaceae). J. Agric. Sci. Technol. A.

[74] Dakole C., Nguefack J., Dongmo Lekagne J.B., Joseph Hubert G., Rene A.U., Somda I., Henry A. (2016). Antifungal Potential of Essential Oils, Aqueous and Ethanol Extracts of Thirteen Plants against Fusarium oxysporum f. Sp Lycopersici and *Phytophtora infestans* (Mont.) de Bary as Major Tomato Pathogens in Cameroon. Int. J. Curr. Sci..

[75] Hubert G.Y.J., Julienne N., Charles D.D., Daniel F., Sandrine P.T., Romain F.F., Henry A.Z. (2013). Antifungal Potential and Phytochemical Analysis of Extracts from Seven Cameroonian Plants against Late Blight Pathogen. Phytophthora Infestans.

[76] Jacquin J., Moureu S., Deweer C., Hakem A., Paguet A.-S., Bonneau N., Bordage S., Dermont C., Sahpaz S., Muchembled J. (2022). Hop (*Humulus lupulus* L.) Specialized Metabolites: Extraction, Purification, Characterization in Different Plant Parts and In Vitro Evaluation of Anti-Oomycete Activities against *Phytophthora infestans*. Agronomy.

[77] Ndala R., Mbega E., Ndakidemi P. (2019). Different Plant Extracts against *Phytophthora infestans* (Mont.) de Bary in Tomato in Vitro. Am. J. Plant Sci..

[78] Bálint J., Turóczi B., Máthé I., Benedek K., Szabó K.-A., Adalbert B. (2014). In Vitro and In Vivo Effect of Poplar Bud (Populi Gemma) Extracts on Late Blight (*Phytophthora infestans*). Acta Univ. Sapientiae Agric. Environ..

[79] Turóczi B., Bakonyi J., Szabó K.-A., Bálint J., Máthé I., Lányi S., Balog A. (2020). In Vitro and In Vivo Effect of Poplar Bud Extracts on *Phytophthora infestans*: A New Effective Biological Method in Potato Late Blight Control. Plants.

[80] Fometu S., Shittu S., Herman R., Ayepa E. (2019). Essential Oils and Their Applications—A Mini Review. Adv. Nutr. Food Sci..

[81] Masango P. (2005). Cleaner Production of Essential Oils by Steam Distillation. J. Clean. Prod..

[82] Gavahian M., Chu Y.-H., Mousavi Khaneghah A. (2019). Recent Advances in Orange Oil Extraction: An Opportunity for the Valorisation of Orange Peel Waste a Review. Int. J. Food Sci. Technol..

[83] Maes C., Bouquillon S., Fauconnier M.-L. (2019). Encapsulation of Essential Oils for the Development of Biosourced Pesticides with Controlled Release: A Review. Molecules.

[84] Slavković F., Bendahmane A. (2023). Floral Phytochemistry: Impact of Volatile Organic Compounds and Nectar Secondary Metabolites on Pollinator Behavior and Health. Chem. Biodivers..

[85] De Clerck C., Genva M., Jijakli M.H., Fauconnier M.-L. (2021). Use of Essential Oils and Volatile Compounds as Biological Control Agents. Foods.

[86] Bourgaud F., Gravot A., Milesi S., Gontier E. (2001). Production of Plant Secondary Metabolites: A Historical Perspective. Plant Sci..

[87] Bhavaniramya S., Vishnupriya S., Al-Aboody M.S., Vijayakumar R., Baskaran D. (2019). Role of Essential Oils in Food Safety: Antimicrobial and Antioxidant Applications. Grain Oil Sci. Technol..

[88] Preedy V.R. (2015). Essential Oils in Food Preservation, Flavor and Safety.

[89] Assadpour E., Can Karaça A., Fasamanesh M., Mahdavi S.A., Shariat-Alavi M., Feng J., Kharazmi M.S., Rehman A., Jafari S.M. (2023). Application of Essential Oils as Natural Biopesticides; Recent Advances. Crit. Rev. Food Sci. Nutr..

[90] Bassolé I.H.N., Juliani H.R. (2012). Essential Oils in Combination and Their Antimicrobial Properties. Molecules.

[91] Faleiro M.L. (2011). The Mode of Antibacterial Action of Essential Oils. Sci. Against Microb. Pathog. Commun. Curr. Res. Technol. Adv..

[92] Fenibo E.O., Ijoma G.N., Matambo T. (2021). Biopesticides in Sustainable Agriculture: A Critical Sustainable Development Driver Governed by Green Chemistry Principles. Front. Sustain. Food Syst..

[93] Najdabbasi N., Mirmajlessi S.M., Dewitte K., Landschoot S., Mänd M., Audenaert K., Ameye M., Haesaert G. (2020). Biocidal Activity of Plant-Derived Compounds against *Phytophthora infestans*: An Alternative Approach to Late Blight Management. Crop Prot..

[94] Soylu E.M., Soylu S., Kurt S. (2006). Antimicrobial Activities of the Essential Oils of Various Plants against Tomato Late Blight Disease Agent *Phytophthora infestans*. Mycopathologia.

[95] Quintanilla P., Rohloff J., Iversen T.-H. (2002). Influence of Essential Oils on *Phytophthora infestans*. Potato Res..

[96] Aksit H., Bayar Y., Simsek S., Ulutas Y. (2022). Chemical Composition and Antifungal Activities of the Essential Oils of Thymus Species (Thymus Pectinatus, Thymus Convolutus, Thymus Vulgaris) Against Plant Pathogens. J. Essent. Oil Bear. Plants.

[97] Carrillo Y.A., Gòmez M.I., Cotes J.M., Ñustez C.E. (2010). Effect of Some Essential Oils on the Growth of *Phytophthora infestans* (Mont.) de Bary under Laboratory Conditions. Agron. Colomb..

[98] Deweer C., Sahmer K., Muchembled J. (2023). Anti-Oomycete Activities from Essential Oils and Their Major Compounds on *Phytophthora infestans*. Environ. Sci. Pollut. Res..

[99] Lu M., Han Z., Yao L. (2013). In Vitro and in Vivo Antimicrobial Efficacy of Essential Oils and Individual Compounds against *Phytophthora parasitica* Var. Nicotianae. J. Appl. Microbiol..

[100] Bi Y., Jiang H., Hausbeck M.K., Hao J.J. (2012). Inhibitory Effects of Essential Oils for Controlling Phytophthora Capsici. Plant Dis..

[101] Maissa B.J., Walid H. (2015). Antifungal Activity of Chemically Different Essential Oils from Wild Tunisian Thymus Spp.. Nat. Prod. Res..

[102] Belay D.W., Asfaw Z., Molla E.L., Kassa B., Kifele H. (2022). Evaluation of Essential Oils Against Potato Late Blight (*Phytophthora infestans* (Mont.) de Bary) at Holleta, Ethiopia. Turk. J. Agric.—Food Sci. Technol..

[103] Vettraino A.M., Zikeli F., Scarascia Mugnozza G., Vinciguerra V., Tabet D., Romagnoli M. (2022). Lignin Nanoparticles Containing Essential Oils for Controlling Phytophthora Cactorum Diseases. For. Pathol..

[104] Thanh V.M., Bui L.M., Bach L.G., Nguyen N.T., Thi H.L., Hoang Thi T.T. (2019). Origanum Majorana L. Essential Oil-Associated Polymeric Nano Dendrimer for Antifungal Activity against *Phytophthora infestans*. Materials.

[105] Pitarokili D., Tzakou O., Loukis A. (2008). Composition of the Essential Oil of Spontaneous Rosmarinus Officinalis from Greece and Antifungal Activity Against Phytopathogenic Fungi. J. Essent. Oil Res..

[106] Amini J., Farhang V., Javadi T., Nazemi J. (2016). Antifungal Effect of Plant Essential Oils on Controlling Phytophthora Species. Plant Pathol. J..

[107] Kamsu F.P.N., Ndondoni Dikongue F.J., Ngouana V., Tchinda E.S., Jiogue M.B., Ambata H.T.A., Tchameni S.N., Sameza M.L., Dongmo Jazet P.M. (2023). Effectiveness of Massep (*Ocimum gratissimum* L.) Essential Oil and Its Nanoemulsion toward Sclerotium Rolfsii, *Phytophthora infestans* and *Alternaria solani*, Pathogens Associated with Tomato Rot Diseases. Biocatal. Agric. Biotechnol..

[108] Al-Mughrabi K.I., Coleman W.K., Vikram A., Poirier R., Jayasuriya K.E. (2013). Effectiveness of Essential Oils and Their Combinations with Aluminum Starch Octenylsuccinate on Potato Storage Pathogens. J. Essent. Oil Bear. Plants.

[109] Banihashemi Z., Abivardi C. (2011). Evaluation of Fungicidal and Fungistatic Activity of Plant Essential Oils towards Plant Pathogenic and Saprophytic Fungi. Phytopathol. Mediterr..

[110] De Clerck C., Maso S.D., Parisi O., Dresen F., Zhiri A., Jijakli M.H. (2020). Screening of Antifungal and Antibacterial Activity of 90 Commercial Essential Oils against 10 Pathogens of Agronomical Importance. Foods.

[111] Mohammadi A., Hashemi M., Hosseini S.M. (2016). Integration between Chitosan and Zataria Multiflora or Cinnamomum Zeylanicum Essential Oil for Controlling Phytophthora Drechsleri, the Causal Agent of Cucumber Fruit Rot. LWT—Food Sci. Technol..

[112] Nana W.L., Eke P., Fokom R., Bakanrga-Via I., Begoude D., Tchana T., Tchameni N.S., Kuate J., Menut C., Fekam Boyom F. (2015). Antimicrobial Activity of Syzygium Aromaticum and Zanthoxylum Xanthoxyloides Essential Oils Against Phytophthora Megakarya. J. Phytopathol..

[113] Hong Z., Talib K.M., Mujtaba K.G., Dabin H., Yahya F., Congying Z., Fukai W. (2021). Antifungal Potential of Cinnamon Essential Oils against Phytophthora Colocasiae Causing Taro Leaf Blight. Chem. Biol. Technol. Agric..

[114] Mugao L.G., Muturi P.W., Gichimu B.M., Njoroge E.K. (2020). In Vitro Control of *Phytophthora infestans* and *Alternaria Solani* Using Crude Extracts and Essential Oils from Selected Plants. Int. J. Agron..

[115] Wang B., Liu F., Li Q., Xu S., Zhao X., Xue P., Feng X. (2019). Antifungal Activity of Zedoary Turmeric Oil against Phytophthora Capsici through Damaging Cell Membrane. Pestic. Biochem. Physiol..

[116] Mugao L.G., Gichimu B.M., Muturi P.W., Njoroge E.K. (2021). Essential Oils as Biocontrol Agents of Early and Late Blight Diseases of Tomato under Greenhouse Conditions. Int. J. Agron..

[117] Kalhoro M.T., Zhang H., Kalhoro G.M., Wang F., Chen T., Faqir Y., Nabi F. (2022). Fungicidal Properties of Ginger (Zingiber Officinale) Essential Oils against Phytophthora Colocasiae. Sci. Rep..

[118] Han X.-B., Zhao J., Cao J.-M., Zhang C.-S. (2019). Essential Oil of *Chrysanthemum indicum* L.: Potential Biocontrol Agent against Plant Pathogen Phytophthora Nicotianae. Environ. Sci. Pollut. Res..

[119] Wang Y., Wei K., Han X., Zhao D., Zheng Y., Chao J., Gou J., Kong F., Zhang C.-S. (2019). The Antifungal Effect of Garlic Essential Oil on Phytophthora Nicotianae and the Inhibitory Component Involved. Biomolecules.

[120] Messgo-Moumene S., Li Y., Bachir K., Houmani Z., Bouznad Z., Chemat F. (2015). Antifungal Power of Citrus Essential Oils against Potato Late Blight Causative Agent. J. Essent. Oil Res..

[121] El-Gamal N.G. (2011). Effect of Orange Essential Oil and Its Constitutes on Late Blight Disease of Potato Plants under Field Conditions. Arch. Phytopathol. Plant Prot..

[122] Tchameni S.N., Mbiakeu S.N., Sameza M.L., Jazet P.M.D., Tchoumbougnang F. (2018). Using Citrus Aurantifolia Essential Oil for the Potential Biocontrol of Colocasia Esculenta (Taro) Leaf Blight Caused by Phytophthora Colocasiae. Environ. Sci. Pollut. Res. Int..

[123] Yang J., Wang Q., Li L., Li P., Yin M., Xu S., Chen Y., Feng X., Wang B. (2022). Chemical Composition and Antifungal Activity of Zanthoxylum Armatum Fruit Essential Oil against Phytophthora Capsici. Molecules.

[124] Wang B., Li P., Yang J., Yong X., Yin M., Chen Y., Feng X., Wang Q. (2022). Inhibition Efficacy of Tetradium Glabrifolium Fruit Essential Oil against Phytophthora Capsici and Potential Mechanism. Ind. Crops Prod..

[125] Snoussi A., Koubaier H., Bouacida S., Essaidi I., Kachouri F., Bouzouita N. (2020). In Vitro Antimicrobial Activity of Carum Carvi L. Seed Essential Oil against Pink Potato Spoilage Flora. Chem. Naissensis.

[126] Benlembarek K., Lograda T., Ramdani M., Figueredo G., Chalard P. (2022). Chemical Composition and Biological Activities of *Anethum graveolens* L. Essential Oil from Algeria. J. Essent. Oil Bear. Plants.

[127] Rubio-Covarrubias O.A., Douches D.S., Hammerschmidt R., daRocha A., Kirk W.W. (2005). Effect of Temperature and Photoperiod on Symptoms Associated with Resistance to *Phytophthora infestans* after Leaf Penetration in Susceptible and Resistant Potato Cultivars. Am. J. Potato Res..

[128] Odilbekov F., Carlson-Nilsson U., Liljeroth E. (2014). Phenotyping Early Blight Resistance in Potato Cultivars and Breeding Clones. Euphytica.

[129] Mihovilovich E., Munive S., Bonierbale M. (2010). Influence of Day-Length and Isolates of *Phytophthora infestans* on Field Resistance to Late Blight of Potato. Theor. Appl. Genet..

[130] Colon L.T., Turkensteen L.J., Prummel W., Budding D.J., Hoogendoorn J. (1995). Durable Resistance to Late Blight (*Phytophthora infestans*) in Old Potato Cultivars. Eur. J. Plant Pathol..

[131] Orłowska E., Fiil A., Kirk H.-G., Llorente B., Cvitanich C. (2012). Differential Gene Induction in Resistant and Susceptible Potato Cultivars at Early Stages of Infection by *Phytophthora infestans*. Plant Cell Rep..

[132] Olanya O.M., Larkin R.P. (2006). Efficacy of Essential Oils and Biopesticides on *Phytophthora infestans* Suppression in Laboratory and Growth Chamber Studies. Biocontrol Sci. Technol..

[133] Gisi U., Walder F., Resheat-Eini Z., Edel D., Sierotzki H. (2011). Changes of Genotype, Sensitivity and Aggressiveness in *Phytophthora infestans* Isolates Collected in European Countries in 1997, 2006 and 2007. J. Phytopathol..

[134] Slusarenko A.J., Patel A., Portz D. (2008). Control of Plant Diseases by Natural Products: Allicin from Garlic as a Case Study. Eur. J. Plant Pathol..

[135] Lubbe A., Verpoorte R. (2011). Cultivation of Medicinal and Aromatic Plants for Specialty Industrial Materials. Ind. Crops Prod..

[136] Cavanagh H. (2007). Antifungal Activity of the Volatile Phase of Essential Oils: A Brief Review. Nat. Prod. Commun..

[137] Werrie P.-Y., Durenne B., Delaplace P., Fauconnier M.-L. (2020). Phytotoxicity of Essential Oils: Opportunities and Constraints for the Development of Biopesticides. A Review. Foods.

[138] Sangwan N.S., Farooqi A.H.A., Shabih F., Sangwan R.S. (2001). Regulation of Essential Oil Production in Plants. Plant Growth Regul..

[139] Moghaddam M., Mehdizadeh L., Grumezescu A.M., Holban A.M. (2017). Chapter 13—Chemistry of Essential Oils and Factors Influencing Their Constituents. Soft Chemistry and Food Fermentation.

[140] Rehman R., Hanif M.A., Mushtaq Z., Al-Sadi A.M. (2016). Biosynthesis of Essential Oils in Aromatic Plants: A Review. Food Rev. Int..

[141] Bakkali F., Averbeck S., Averbeck D., Idaomar M. (2008). Biological Effects of Essential Oils—A Review. Food Chem. Toxicol..

[142] Ashour M., Wink M., Gershenzon J. (2010). Biochemistry of Terpenoids: Monoterpenes, Sesquiterpenes and Diterpenes. Annual Plant Reviews Volume 40: Biochemistry of Plant Secondary Metabolism.

[143] Dixon R.A., Achnine L., Kota P., Liu C.-J., Reddy M.S.S., Wang L. (2002). The Phenylpropanoid Pathway and Plant Defence—A Genomics Perspective. Mol. Plant Pathol..

[144] De Cássia da Silveira e Sá R., Andrade L.N., Dos Reis Barreto de Oliveira R., De Sousa D.P. (2014). A Review on Anti-Inflammatory Activity of Phenylpropanoids Found in Essential Oils. Molecules.

[145] Barra A. (2009). Factors Affecting Chemical Variability of Essential Oils: A Review of Recent Developments. Nat. Prod. Commun..

[146] Novak J., Draxler L., Göhler I., Franz C.M. (2005). Essential Oil Composition of Vitex Agnus-Castus—Comparison of Accessions and Different Plant Organs. Flavour Fragr. J..

[147] Boira H., Blanquer A. (1998). Environmental Factors Affecting Chemical Variability of Essential Oils in *Thymus piperella* L.. Biochem. Syst. Ecol..

[148] Melito S., Petretto G.L., Podani J., Foddai M., Maldini M., Chessa M., Pintore G. (2016). Altitude and Climate Influence Helichrysum Italicum Subsp. Microphyllum Essential Oils Composition. Ind. Crops Prod..

[149] Llorens-Molina J.A., Vacas S. (2017). Effect of Drought Stress on Essential Oil Composition of *Thymus vulgaris* L. (Chemotype 1, 8-Cineole) from Wild Populations of Eastern Iberian Peninsula. J. Essent. Oil Res..

[150] Najar B., Pistelli L., Ferri B., Angelini L.G., Tavarini S. (2021). Crop Yield and Essential Oil Composition of Two Thymus Vulgaris Chemotypes along Three Years of Organic Cultivation in a Hilly Area of Central Italy. Molecules.

[151] Yang L., Wen K.-S., Ruan X., Zhao Y.-X., Wei F., Wang Q. (2018). Response of Plant Secondary Metabolites to Environmental Factors. Molecules.

[152] Letchamo W., Ward W., Heard B., Heard D. (2004). Essential Oil of *Valeriana officinalis* L. Cultivars and Their Antimicrobial Activity As Influenced by Harvesting Time under Commercial Organic Cultivation. J. Agric. Food Chem..

[153] Zouari N. (2013). Essential Oils Chemotypes: A Less Known Side. Med. Aromat. Plants.

[154] Kaloustian J., Abou L., Mikail C., Amiot M.J., Portugal H. (2005). Southern French Thyme Oils: Chromatographic Study of Chemotypes. J. Sci. Food Agric..

[155] Lis-Balchin M., Deans S.G., Eaglesham E. (1998). Relationship between Bioactivity and Chemical Composition of Commercial Essential Oils. Flavour Fragr. J..

[156] Marriott P.J., Shellie R., Cornwell C. (2001). Gas Chromatographic Technologies for the Analysis of Essential Oils. J. Chromatogr. A.

[157] Pérez-Izquierdo C., Serrano-Pérez P., del Carmen Rodríguez-Molina M. (2022). Chemical Composition, Antifungal and Phytotoxic Activities of Cistus Ladanifer L. Essential Oil and Hydrolate. Biocatal. Agric. Biotechnol..

[158] Dan Y., Liu H.-Y., Gao W.-W., Chen S.-L. (2010). Activities of Essential Oils from *Asarum heterotropoides* Var. Mandshuricum against Five Phytopathogens. Crop Prot..

[159] Verdeguer M., Sánchez-Moreiras A.M., Araniti F. (2020). Phytotoxic Effects and Mechanism of Action of Essential Oils and Terpenoids. Plants.

[160] Taheri P., Soweizy M., Tarighi S. (2023). Application of Essential Oils to Control Some Important Fungi and Bacteria Pathogenic on Cereals. J. Nat. Pestic. Res..

[161] Samuels L., Kunst L., Jetter R. (2008). Sealing Plant Surfaces: Cuticular Wax Formation by Epidermal Cells. Annu. Rev. Plant Biol..

[162] Cristani M., D’Arrigo M., Mandalari G., Castelli F., Sarpietro M.G., Micieli D., Venuti V., Bisignano G., Saija A., Trombetta D. (2007). Interaction of Four Monoterpenes Contained in Essential Oils with Model Membranes: Implications for Their Antibacterial Activity. J Agric. Food Chem..

[163] Brennan T.C.R., Krï¿½mer J.O., Nielsen L.K. (2013). Physiological and Transcriptional Responses of Saccharomyces Cerevisiae to D-Limonene Show Changes to the Cell Wall but Not to the Plasma Membrane. Appl. Environ. Microbiol..

[164] Ziogas B.N., Markoglou A.N., Theodosiou D.I., Anagnostou A., Boutopoulou S. (2006). A High Multi-Drug Resistance to Chemically Unrelated Oomycete Fungicides in *Phytophthora infestans*. Eur. J. Plant Pathol..

[165] Mei X., Liu Y., Huang H., Du F., Huang L., Wu J., Li Y., Zhu S., Yang M. (2019). Benzothiazole Inhibits the Growth of Phytophthora Capsici through Inducing Apoptosis and Suppressing Stress Responses and Metabolic Detoxification. Pestic. Biochem. Physiol..

[166] Ben Jabeur M., Somai-Jemmali L., Hamada W. (2017). Thyme Essential Oil as an Alternative Mechanism: Biofungicide-causing Sensitivity of Mycosphaerella Graminicola. J. Appl. Microbiol..

[167] Tariq S., Wani S., Rasool W., Shafi K., Bhat M.A., Prabhakar A., Shalla A.H., Rather M.A. (2019). A Comprehensive Review of the Antibacterial, Antifungal and Antiviral Potential of Essential Oils and Their Chemical Constituents against Drug-Resistant Microbial Pathogens. Microb. Pathog..

[168] Wang Y., Zhang C., Liang J., Wu L., Gao W., Jiang J. (2020). Iturin A Extracted From Bacillus Subtilis WL-2 Affects *Phytophthora infestans* via Cell Structure Disruption, Oxidative Stress, and Energy Supply Dysfunction. Front. Microbiol..

[169] Yu L.M. (1995). Elicitins from Phytophthora and Basic Resistance in Tobacco. Proc. Natl. Acad. Sci. USA.

[170] Vandana V.V., Suseela Bhai R., Ramakrishnan Nair R., Azeez S. (2019). Role of Cell Wall and Cell Membrane Integrity in Imparting Defense Response against Phytophthora Capsici in Black Pepper (*Piper nigrum* L.). Eur. J. Plant Pathol..

[171] Jing C., Gou J., Han X., Wu Q., Zhang C. (2017). In Vitro and in Vivo Activities of Eugenol against Tobacco Black Shank Caused by Phytophthora Nicotianae. Pestic. Biochem. Physiol..

[172] Gill S.S., Tuteja N. (2010). Reactive Oxygen Species and Antioxidant Machinery in Abiotic Stress Tolerance in Crop Plants. Plant Physiol. Biochem..

[173] Guimarães A.C., Meireles L.M., Lemos M.F., Guimarães M.C.C., Endringer D.C., Fronza M., Scherer R. (2019). Antibacterial Activity of Terpenes and Terpenoids Present in Essential Oils. Molecules.

[174] Tang T., Zhong W., Yang L., He M., Jiang S., Yin D., Guo J., Gao Z. (2024). In Vitro and in Vivo Anti-Oomycetes Activities and Mechanisms of Linalool against Saprolegnia Ferax. Aquaculture.

[175] Gao T., Zhou H., Zhou W., Hu L., Chen J., Shi Z. (2016). The Fungicidal Activity of Thymol against Fusarium Graminearum via Inducing Lipid Peroxidation and Disrupting Ergosterol Biosynthesis. Molecules.

[176] Zhang J., Hao Y., Lu H., Li P., Chen J., Shi Z., Xie Y., Mo H., Hu L. (2022). Nano-Thymol Emulsion Inhibits Botrytis Cinerea to Control Postharvest Gray Mold on Tomato Fruit. Agronomy.

[177] Pei S., Liu R., Gao H., Chen H., Wu W., Fang X., Han Y. (2020). Inhibitory Effect and Possible Mechanism of Carvacrol against Colletotrichum Fructicola. Postharvest Biol. Technol..

[178] Crowley L.C., Scott A.P., Marfell B.J., Boughaba J.A., Chojnowski G., Waterhouse N.J. (2016). Measuring Cell Death by Propidium Iodide Uptake and Flow Cytometry. Cold Spring Harb. Protoc..

[179] Davis D.J., Burlak C., Money N.P. (2000). Osmotic Pressure of Fungal Compatible Osmolytes. Mycol. Res..

[180] Lippincott-Schwartz J., Phair R.D. (2010). Lipids and Cholesterol as Regulators of Traffic in the Endomembrane System. Annu. Rev. Biophys..

[181] Han Y., Sun Z., Chen W. (2020). Antimicrobial Susceptibility and Antibacterial Mechanism of Limonene against Listeria Monocytogenes. Molecules.

[182] Zhang Y., Feng R., Li L., Zhou X., Li Z., Jia R., Song X., Zou Y., Yin L., He C. (2018). The Antibacterial Mechanism of Terpinen-4-Ol Against Streptococcus Agalactiae. Curr. Microbiol..

[183] Vila R., Freixa B., Cañigueral S. (2013). Antifungal Compounds from Plants. Phytochemical Resources for Medicine and Agriculture.

[184] Song W., Yin Z., Lu X., Shen D., Dou D. (2023). Plant Secondary Metabolite Citral Interferes with Phytophthora Capsici Virulence by Manipulating the Expression of Effector Genes. Mol. Plant Pathol..

[185] Ghalem B.R., Choudhary D.K., Varma A., Tuteja N. (2016). Essential Oils as Antimicrobial Agents against Some Important Plant Pathogenic Bacteria and Fungi. Plant-Microbe Interaction: An Approach to Sustainable Agriculture.

[186] Ngegba P.M., Cui G., Khalid M.Z., Zhong G. (2022). Use of Botanical Pesticides in Agriculture as an Alternative to Synthetic Pesticides. Agriculture.

[187] Turek C., Stintzing F.C. (2013). Stability of Essential Oils: A Review. Compr. Rev. Food Sci. Food Saf..

[188] Lucia A., Guzmán E. (2021). Emulsions Containing Essential Oils, Their Components or Volatile Semiochemicals as Promising Tools for Insect Pest and Pathogen Management. Adv. Colloid Interface Sci..

[189] Dunan L., Malanga T., Benhamou S., Papaiconomou N., Desneux N., Lavoir A.-V., Michel T. (2023). Effects of Essential Oil-Based Formulation on Biopesticide Activity. Ind. Crops Prod..

[190] Pavoni L., Perinelli D.R., Bonacucina G., Cespi M., Palmieri G.F. (2020). An Overview of Micro- and Nanoemulsions as Vehicles for Essential Oils: Formulation, Preparation and Stability. Nanomaterials.

[191] Tadros T., Izquierdo P., Esquena J., Solans C. (2004). Formation and Stability of Nano-Emulsions. Adv. Colloid Interface Sci..

[192] Anjali C.H., Sharma Y., Mukherjee A., Chandrasekaran N. (2012). Neem Oil (*Azadirachta indica*) Nanoemulsion—A Potent Larvicidal Agent against Culex Quinquefasciatus. Pest Manag. Sci..

[193] Wu J.-E., Lin J., Zhong Q. (2014). Physical and Antimicrobial Characteristics of Thyme Oil Emulsified with Soluble Soybean Polysaccharide. Food Hydrocoll..

[194] Sugumar S., Singh S., Mukherjee A., Chandrasekaran N. (2016). Nanoemulsion of Orange Oil with Non Ionic Surfactant Produced Emulsion Using Ultrasonication Technique: Evaluating against Food Spoilage Yeast. Appl. Nanosci..

[195] Tang C., Li Y., Pun J., Mohamed Osman A.S., Tam K.C. (2019). Polydopamine Microcapsules from Cellulose Nanocrystal Stabilized Pickering Emulsions for Essential Oil and Pesticide Encapsulation. Colloids Surf. A Physicochem. Eng. Asp..

[196] Rajkumar V., Gunasekaran C., Paul C.A., Dharmaraj J. (2020). Development of Encapsulated Peppermint Essential Oil in Chitosan Nanoparticles: Characterization and Biological Efficacy against Stored-Grain Pest Control. Pestic. Biochem. Physiol..

[197] Campos E.V.R., Proença P.L.F., Oliveira J.L., Pereira A.E.S., de Morais Ribeiro L.N., Fernandes F.O., Gonçalves K.C., Polanczyk R.A., Pasquoto-Stigliani T., Lima R. (2018). Carvacrol and Linalool Co-Loaded in β-Cyclodextrin-Grafted Chitosan Nanoparticles as Sustainable Biopesticide Aiming Pest Control. Sci. Rep..

[198] Ez-Zoubi A., Ez Zoubi Y., Bentata F., El-Mrabet A., Ben Tahir C., Labhilili M., Farah A. (2023). Preparation and Characterization of a Biopesticide Based on *Artemisia Herba-Alba* Essential Oil Encapsulated with Succinic Acid-Modified Beta-Cyclodextrin. J. Chem..

[199] López M.D., Cantó-Tejero M., Pascual-Villalobos M.J. (2021). New Insights Into Biopesticides: Solid and Liquid Formulations of Essential Oils and Derivatives. Front. Agron..

[200] Barradas T.N., de Holanda e Silva K.G. (2021). Nanoemulsions of Essential Oils to Improve Solubility, Stability and Permeability: A Review. Environ. Chem. Lett..

[201] Hardham A.R. (2001). The Cell Biology behind Phytophthora Pathogenicity. Australas. Plant Pathol..

[202] Isman M.B. (2000). Plant Essential Oils for Pest and Disease Management. Crop Prot..

[203] Kazan K., Gardiner D.M. (2017). Targeting Pathogen Sterols: Defence and Counterdefence?. PLoS Pathog..

[204] Gaulin E., Bottin A., Dumas B. (2010). Sterol Biosynthesis in Oomycete Pathogens. Plant Signal. Behav..

[205] Wang W., Liu X., Govers F. (2021). The Mysterious Route of Sterols in Oomycetes. PLoS Pathog..

[206] Wang K., Senthil-Kumar M., Ryu C.-M., Kang L., Mysore K.S. (2012). Phytosterols Play a Key Role in Plant Innate Immunity against Bacterial Pathogens by Regulating Nutrient Efflux into the Apoplast. Plant Physiol..

[207] Amborabé B.-E., Rossard S., Pérault J.-M., Roblin G. (2003). Specific Perception of Ergosterol by Plant Cells. Comptes Rendus Biol..

[208] Dahlin P., Srivastava V., Ekengren S., McKee L.S., Bulone V. (2017). Comparative Analysis of Sterol Acquisition in the Oomycetes Saprolegnia Parasitica and *Phytophthora infestans*. PLoS ONE.

[209] Giunti G., Benelli G., Palmeri V., Laudani F., Ricupero M., Ricciardi R., Maggi F., Lucchi A., Guedes R.N.C., Desneux N. (2022). Non-Target Effects of Essential Oil-Based Biopesticides for Crop Protection: Impact on Natural Enemies, Pollinators, and Soil Invertebrates. Biol. Control.

[210] Zhang S., Wang Y., Cai J., Liu D., Yan Y., Zhang H., Li L., Wang X., Xiang W., Zhang J. (2022). Discovery of Febrifugine with Specific Anti-Phytophthora Oomycete Activity Isolated from Dichroa Febrifuga Lour. Ind. Crops Prod..

[211] Stanley J., Preetha G., Stanley J., Preetha G. (2016). Pesticide Toxicity to Microorganisms: Exposure, Toxicity and Risk Assessment Methodologies. Pesticide Toxicity to Non-Target Organisms: Exposure, Toxicity and Risk Assessment Methodologies.

[212] Carlile B. (2006). Pesticide Selectivity, Health and the Environment.

[213] Alengebawy A., Abdelkhalek S.T., Qureshi S.R., Wang M.-Q. (2021). Heavy Metals and Pesticides Toxicity in Agricultural Soil and Plants: Ecological Risks and Human Health Implications. Toxics.

[214] Michael O.T., Adebayo O. (2017). Plant Essential Oil: An Alternative to Emerging Multidrug Resistant Pathogens. J. Microbiol. Exp..

[215] Saltos-Rezabala L.A., Silveira P.R.D., Tavares D.G., Moreira S.I., Magalhães T.A., Botelho D.M.D.S., Alves E. (2022). Thyme Essential Oil Reduces Disease Severity and Induces Resistance against Alternaria Linariae in Tomato Plants. Horticulturae.

[216] Kesraoui S., Andrés M.F., Berrocal-Lobo M., Soudani S., Gonzalez-Coloma A. (2022). Direct and Indirect Effects of Essential Oils for Sustainable Crop Protection. Plants.

[217] Rienth M., Crovadore J., Ghaffari S., Lefort F. (2019). Oregano Essential Oil Vapour Prevents Plasmopara Viticola Infection in Grapevine (Vitis Vinifera) and Primes Plant Immunity Mechanisms. PLoS ONE.

[218] Lengnick L. (2015). The Vulnerability of the US Food System to Climate Change. J. Environ. Stud. Sci..

[219] Malik A., Suryapani S., Ahmad J. (2011). Chemical Vs Organic Cultivation of Medicinal and Aromatic Plants: The Choice Is Clear. Int. J. Med. Aromat. Plants.

[220] Balog A., Hartel T., Loxdale H.D., Wilson K. (2017). Differences in the Progress of the Biopesticide Revolution between the EU and Other Major Crop-Growing Regions. Pest Manag. Sci..

[221] Pavela R., Benelli G. (2016). Essential Oils as Ecofriendly Biopesticides? Challenges and Constraints. Trends Plant Sci..

[222] Gounaris Y. (2010). Biotechnology for the Production of Essential Oils, Flavours and Volatile Isolates. A Review. Flavour Fragr. J..

[223] Teper-Bamnolker P., Dudai N., Fischer R., Belausov E., Zemach H., Shoseyov O., Eshel D. (2010). Mint Essential Oil Can Induce or Inhibit Potato Sprouting by Differential Alteration of Apical Meristem. Planta.

[224] Thoma J., Zheljazkov V.D. (2022). Sprout Suppressants in Potato Storage: Conventional Options and Promising Essential Oils—A Review. Sustainability.

[225] Kundu R., Dutta D., Nanda M.K., Chakrabarty A. (2021). Near Real Time Monitoring of Potato Late Blight Disease Severity Using Field Based Hyperspectral Observation. Smart Agric. Technol..

[226] Laothawornkitkul J., Jansen R.M.C., Smid H.M., Bouwmeester H.J., Muller J., van Bruggen A.H.C. (2010). Volatile Organic Compounds as a Diagnostic Marker of Late Blight Infected Potato Plants: A Pilot Study. Crop Prot..

[227] Popp J., Pető K., Nagy J. (2013). Pesticide Productivity and Food Security. A Review. Agron. Sustain. Dev..

[228] Dassanayake M.K., Chong C.H., Khoo T.-J., Figiel A., Szumny A., Choo C.M. (2021). Synergistic Field Crop Pest Management Properties of Plant-Derived Essential Oils in Combination with Synthetic Pesticides and Bioactive Molecules: A Review. Foods.

